# Dynamic Proton arc therapy for paediatric parameningeal rhabdomyosarcoma

**DOI:** 10.1002/acm2.70867

**Published:** 2026-10-20

**Authors:** Andreas Havsgård Handeland, Hardev Singh Grewal, Camilla Grindeland Boer, Daniel J. Indelicato, Kristian Smeland Ytre‐Hauge, Helge Henjum, Camilla Hanquist Stokkevåg

**Affiliations:** ^1^ Cancer Clinic Haukeland University Hospital Bergen Norway; ^2^ Department of Physics and Technology University of Bergen Bergen Norway; ^3^ Department of Radiation Oncology University of Florida College of Medicine Jacksonville Florida USA; ^4^ University of Florida Health Proton Therapy Institute Jacksonville Florida USA

**Keywords:** linear energy transfer, linear energy transfer‐optimization, pediatric, proton arc therapy, Proton therapy, rhabdomyosarcoma

## Abstract

**Background:**

Parameningeal rhabdomyosarcomas (PM‐RMS) are pediatric tumors with a high risk of local failure and severe toxicity. Intensity modulated proton therapy (IMPT) offers improved healthy tissue sparing compared to conventional photon‐based radiotherapy, and proton arc therapy (PAT) might offer further improvement in healthy tissue sparing for these patients, thus emphasizing the need for systematic studies specifically focusing on PM‐RMS patients. Additionally, the relative biological effectiveness (RBE) of protons is known to vary with linear energy transfer (LET).

**Purpose:**

The aim of this study was to investigate the potential of LET‐optimized PAT for PM‐RMS treatment.

**Methods:**

Volumetric modulated arc therapy (VMAT) plans, IMPT and PAT plans, both with and without LET optimization were made for 13 PM‐RMS patients using Raystation version 2023B. The plans were evaluated based on the achievement of clinical goals, homogeneity index, delivery time, robustness, LET and variable RBE‐weighted dose within the target and selected OARs.

**Results:**

LET optimization increased the mean LET in the target from 2.5 keV/µm [2.1, 2.8] to 3.4 keV/µm [2.7, 4.1] for IMPT and from 2.8 keV/µm [2.5, 3.1] to 3.4 keV/µm [2.7, 4.0] for PAT with minor changes in dose distributions. Target coverage and OAR doses were comparable between IMPT and PAT, while both had an expected improvement in OAR sparing compared to VMAT. Delivery times were 134.5 [95.3, 203.7] seconds for the IMPT plans and 197.4 [138.9, 244.6] seconds for the PAT plans before LET optimization, and were for both modalities reduced by around 2–5 seconds after LET optimization.

**Conclusions:**

The dose distribution was similar across PAT and IMPT in this study, and superior compared to VMAT. LET optimization in the proton plans achieved LET improvements in both target and OARs without compromising dose.

## 1 INTRODUCTION

Parameningeal rhabdomyosarcomas (PM‐RMS) are the most common pediatric soft tissue sarcoma and often present as large tumors near critical brain structures and the skull base. Although the majority of pediatric rhabdomyosarcoma patients achieve long‐term survival, with 5‐year overall survival rates typically around 80%,^1^, ^2^, ^3^ children with tumors of the parameningeal sites remain vulnerable with a high risk of local failure.^2^, ^4^ In addition to suboptimal disease control, grade 3–4 toxicity is a considerable concern for these patients with reported rates at 10% or higher.^1^, ^3^ Both acute toxicities such as febrile neutropenia and lymphopenia^5^ and late toxicities in the form of reduced facial growth^6^ are experienced by these patients, making them candidates for proton therapy.^1^, ^7^

Proton therapy offers consistent normal tissue sparing compared to conventional photon‐based radiotherapy^8^, ^9^ and comparative studies of patients treated with passively scattered protons have shown improved dose distributions with protons for PM‐RMS patients.^7^, ^10^ Since then, proton technology has evolved and intensity modulated proton therapy (IMPT) allows more accurate targeting of dose.^11^, ^12^ Emerging proton arc therapy (PAT) has gained increased research focus in later years^13^ and has the potential to further enhance normal tissue sparing for these patients,^14^, ^15^ making PAT a promising option for challenging tumor locations, such as PM‐RMS. By utilizing proton arcs instead of discrete beams as with IMPT, the low dose volume is expanded but the integral dose may be decreased.^16^ Since PM‐RMS have several organs at risk (OAR) both proximal and distant to the target, the overall OAR dose and associated toxicity risk may be lowered through the use of PAT. However, this dose benefit needs to be balanced against a potential increase in low dose volumes emphasizing the need of quantifying the potential of PAT for these patients. A comparison of IMPT and PAT for PM‐RMS patients will therefore give insight into both advantages and disadvantages with both modalities.

Where a constant relative biological effectiveness (RBE) is assumed clinically, the proton RBE is known to vary with linear energy transfer (LET) among other factors.^17^, ^18^ Several clinical studies suggest a relationship between increased LET and toxicity^19^, ^20^ although results from the literature vary considerably.^21^ As recently outlined in a review by McIntyre et al.,^22^ head and neck tumor patients may be at particular risk of LET‐effects due to the presence of LET hotspots from asymmetric beam arrangements and may therefore be strong candidates for the utilization of LET optimization. Incorporating LET‐based criteria in proton planning could in theory enhance the biological effectiveness of protons within the target volume and lower the effectiveness in OARs. This could effectively improve local control and risk of toxicity. In this manner, LET optimization of PAT has specifically demonstrated the ability to lower the LET in OARs and increase LET in the target quite effectively compared to IMPT^23^, ^24^ which makes LET optimization and PAT both attractive options for use in PM‐RMS patients.

This study aimed to investigate the normal tissue sparing potential of PAT compared to IMPT and benchmark both against VMAT plans for PM‐RMS patients. In addition, the aim was to assess the impact of LET optimization on both PAT and IMPT.

## 2 METHODS

### 2.1 Patient material

The patient material consisted of 13 anonymized pediatric patients previously treated for PM‐RMS at [ANONYMIZED]. The patients were prescribed a *D*_99%_ (dose to 99% of the volume) of 36 Gy(RBE) to the clinical target volume (CTV), encompassing the gross tumor volume (GTV) and tumor bed, with a subsequent boost of between 14.4 Gy(RBE) and 23.4 Gy(RBE) based on tumor size to the GTV alone. Total prescribed doses were 50.4 Gy(RBE) for ten patients, 55.8 Gy(RBE) for two patients and 59.4 for one patient (Table A1). GTVs ranged between 4.07–163.31 cm^3^ and CTVs ranged between 20.6–332.57 cm^3^ with varying tumor locations (Some examples are given in Figure A1). The use of the patients was approved by the [ANONYMIZED].

### 2.2 Treatment planning

The patients were replanned with volumetric modulated arc therapy (VMAT) with photons, IMPT, and PAT, both with and without LET optimization. All plans were made in Raystation 2023B Research Version (RaySearch Laboratories, Stockholm, Sweden). The VMAT plans, and half of the proton plans (both IMPT and PAT) were planned at [Anonymized institution 1] while the remaining half of protons plans were planned at [Anonymized institution 2]. To ensure standardization in treatment plan quality between institutions the same clinical constraints and prioritization were followed, and the first patient was planned together by planners at both institutions over a digital meeting. Additionally, spot spacing, energy layer spacing, and gantry angle spacing were all kept consistent between the two institutions. Dose statistics for OARs and target volumes for the two institutions are given in Tables A2‐A3.

The VMAT plans were generated primarily using two full arcs (Figure 1a), aiming to avoid or minimize entry dose through the contralateral healthy tissues. For smaller target volumes, two partial arcs were used. In cases where the target volume was close to the skin surface, a bolus was added to ensure adequate dose coverage to the entire tumor. The IMPT plans used between 3–5 fields avoiding field angles too close together and avoiding placing distal ends in OARs, particularly the brainstem, if possible (Figure 1b). The PAT plans were dynamic arc plans with a single energy layer per beam angle. The PAT plans applied two semi‐arcs in opposite directions wide enough to cover the target volume (Figure 1c). To spare opposing healthy tissue, the two arcs covered similar or the same angles for shallow tumors. The Early Layer and Spot Assign (ELSA) algorithm was used to optimize the PAT plans.^25^ All the proton plans were robustly optimized with respect to the CTV with 3.5% range uncertainty and 3 mm isotropic shifts in all spatial directions. Plans were considered clinically acceptable if they had a pass‐rate of 100% with *D*_95%_ > 95% to the target volume.

**FIGURE 1:**
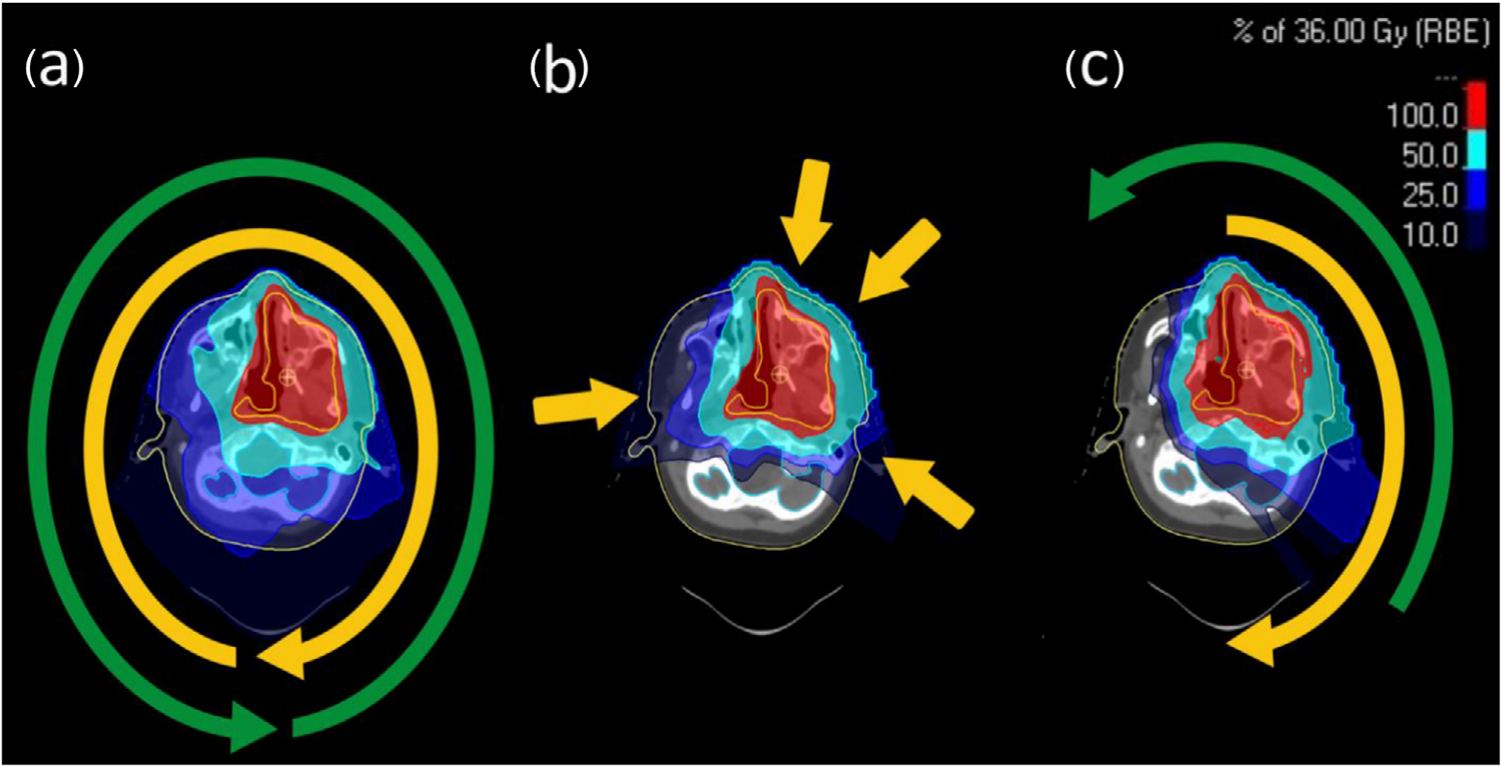
An example patient with a shallow RMS tumour treatment planned with: a) VMAT consisting of two full coplanar arcs in opposite rotational directions, b) 4‐field IMPT and c) PAT consisting of two coplanar partial arcs covering similar angles. The CTV is shown in yellow. Dose from initial prescription 36 Gy(RBE) is shown.

In the first planning phase, optimization was based on dose criteria only. Subsequently, in the second planning phase for the LET optimized plans, LET criteria were added to the cost function in order to investigate if the LET could be lowered in OARs and increased in the target volume. LET was defined as dose‐averaged LET (referred to simply as LET) from primary and secondary protons, scored in water. A maximum LET of 2 keV/µm in volumes receiving dose of 15 Gy(RBE) or higher for the initial plan and 5–8 Gy(RBE) for the boost plan was set as the goal to the brainstem, optic chiasm, optic nerves, cochleae and spinal cord. A goal of 5 keV/µm minimum LET in the GTV was also set. These dose and LET levels were chosen pragmatically to encourage the optimizer to improve the LET without while preventing LET objectives from dominating the optimization function and worsening the dose distributions. Different dose thresholds were chosen for the initial plan and the boost plan to maintain a similar percentage of 30–40% of the prescribed dose for both sub‐plans. The LET criteria were weighted at 1 or below, while OARs were between 1 and 100 and target volumes were generally weighted between 1000–10000.

### 2.3 Plan evaluation

Plan quality was evaluated with dose‐volume histograms (DVH) and the ability of plans to meet clinical criteria for OARs. Clinical criteria were based on [Anonymized institution 2] clinical guidelines (Table 1) and consisted of dose goals and dose constraints, where both should be met but the latter outweighs the former. The brainstem constraints were the only constraints that were prioritized over target coverage. Dose statistics related to the individual clinical goals were thus compared between all five techniques. We also investigated the homogeneity index (HI) as an assessment of target coverage for all modalities. We used the definition of HI proposed by Wu et al.^26^ (Equation A1). The robustness of target coverage was assessed for all proton modalities, where coverage of the CTV was considered for the initial plan, while GTV coverage accounted for both the initial plan and the boost plan, but was assessed individually for the two sub‐plans. A combination of range and setup shifts were considered for a total of 45 scenarios. Relative delivery time between the proton modalities was estimated using the arc trajectory optimization method (ATOM) for the dynamic arc plans, where default values set by ATOM were used for gantry velocity, acceleration, spot delivery time, spot switching time and energy layer switching time.^27^ The IMPT plans used the Ruckig package^28^ to estimate gantry trajectories, which is the same package utilized by ATOM, and incorporated the same default parameters as ATOM. Mean dose to the brain and to the body was used as a surrogate for integral dose from the different modalities. A series of uncorrected Wilcoxon signed rank tests were used primarily to demonstrate how systematically different dose, LET, delivery times, number of spots and energy layers were between modalities and while the differences are described as statistically significant in the manuscript, they should not be emphasized as statistical evidence of a significant difference between the two modalities compared.

**TABLE 1:** [Anonymized institution 2] clinical dose criteria utilized in the planning. Certain regions of interest (ROI) have both a defined goal and hard constraint while others only have a soft constraint or preference. *D*_0.1cc_ is the dose at 1 cm^3^ volume.

ROI	Dose Metric	Constraint	Goal
Left Retina	*D*_0.1cc_	55 Gy(RBE)	50 Gy(RBE)
Right Retina	*D*_0.1cc_	55 Gy(RBE)	50 Gy(RBE)
Pituitary	Mean Dose	30 Gy(RBE)	
Hypothalamus	Mean Dose	5 Gy(RBE)	
Corpus Callosum	Mean Dose	24 Gy(RBE)	
Right Optical Nerve	*D*_0.1cc_	60 Gy(RBE)	55 Gy(RBE)
Left Optical Nerve	*D*_0.1cc_	60 Gy(RBE)	55 Gy(RBE)
Right Cornea	*D*_0.1cc_	35 Gy(RBE)	
Left Cornea	*D*_0.1cc_	35 Gy(RBE)	
Brainstem	*D*_0.1cc_	58 Gy(RBE)	56.6 Gy(RBE)
Brainstem	*D*_10_	56 Gy(RBE)	55.4 Gy(RBE)
Brainstem	*D*_50_	54 Gy(RBE)	52.4 Gy(RBE)
Brainstem Core	Max Dose	56.1 Gy(RBE)	
Optic Chiasm	*D*_0.1cc_	60 Gy(RBE)	55 Gy(RBE)
Right Temporal Lobe	*V*_20Gy_	10%	
Left Temporal Lobe	*V*_20Gy_	10%	
Right Hippocampus	Mean Dose	20 Gy(RBE)	
Left Hippocampus	Mean Dose	20 Gy(RBE)	
Right Hippocampus Head	Mean Dose	5 Gy(RBE)	
Left Hippocampus Head	Mean Dose	5 Gy(RBE)	
Right Hippocampus Tail	Mean Dose	20 Gy(RBE)	
Left Hippocampus Tail	Mean Dose	20 Gy(RBE)	
Right Lacrimal Gland	Mean Dose	26 Gy(RBE)	10 Gy(RBE)
Left Lacrimal Gland	Mean Dose	26 Gy(RBE)	10 Gy(RBE)
Right Cochlea	Mean Dose	36 Gy(RBE)	30 Gy(RBE)
Left Cochlea	Mean Dose	36 Gy(RBE)	30 Gy(RBE)
Spinal Cord	*V*_50.4_ _Gy_	5 cm^3^	
Spinal Cord	0.1 cc Dose	52.2 Gy(RBE)	50.4 Gy(RBE)
Right Lens	Mean Dose	2 Gy(RBE)	
Left Lens	Mean Dose	2 Gy(RBE)	
CTV	*D*_99%_	100%	
CTV	*V*_99%_	100%	
GTV	*D*_99%_	100%	
GTV	*V*_99%_	100%	

Dose was evaluated across all techniques comparing the absorbed dose for VMAT and RBE_1.1_‐weighted dose for the proton modalities. LET in OARs and the target volume was also compared between the proton plans, evaluated individually for the initial plan and the boost plan. Additionally, the combined effect of dose and LET was considered through the use of variable RBE (RBE_VAR_) models where the Lyngholm model (LYN)^29^ was utilized since it is based on a large amount of proton data (Equation A2‐A4). Additionally, the Unkelbach model or LET‐weighted dose (LWD)^30^ (Equation A7) and the McNamara (MCN) model^31^ (Equation A1, A5–A6) were used since these are models commonly used in the literature. LWD was chosen because it is independent of α/β and gives a direct relationship dependent only on dose and LET, while LYN and MCN are phenomenological RBE models with different assumptions and fitted data, with the former resulting in slightly higher RBE estimates than the latter. For LWD the scaling parameter *c* was set to 0.04.^30^ The α/β values applied in the LYN and MCN models were 2.0 Gy for healthy brain tissue and 2.8 Gy for the CTV.^32^

## 3 RESULTS

PAT had similar or slightly reduced OAR doses compared to IMPT, but none were statistically significant from a Wilcoxon signed rank test (Figure 2, Table 2). As an example, the median patient had mean dose to the ipsilateral cochlea of 38.27 Gy(RBE) [Range: 9.32, 51.28] and 40.61 Gy(RBE) [10.91, 51.80] for IMPT, and 36.35 Gy(RBE) [8.76, 53.84] and 39.85 Gy(RBE) [12.29, 54.24] for PAT, both referring to non‐LET optimized and LET‐optimized plans, respectively (Figure 2, Table 2). LET optimization caused marginal change in RBE_1.1_‐weighted doses with the LET‐optimized IMPT plan showing a slight but statistically significant increase in mean dose to the body, ipsilateral cochlea and pituitary gland (Figure 2, Table A4). Nominal GTV coverage was similar across all modalities, but the median GTV dose was overall marginally increased with PAT (Figure 2, Table 2, Figure A2, Table A4). LET optimization similarly resulted in a slight increase in median dose to the GTV for both proton modalities (Figure 2, Table A4). Robustness was, however, slightly reduced with LET optimization, with 100% [100%, 100%] pass rate in *D*_95%_ > 95% for IMPT and 100% [98%, 100%] for PAT prior to LET optimization, reduced to 91–94% [67%, 100%] for IMPT and 92–93% [67%, 100%] for PAT after LET optimization, respectively (Table 3). Homogeneity in the GTV was improved with VMAT over both proton modalities, and IMPT was slightly superior to PAT. LET optimization resulted in a slight loss of homogeneity for the IMPT plans (Figure 2, Table 2, Table A4). The VMAT‐plans had statistically significantly higher doses to most OARs including the brainstem, brain and body compared to both proton modalities, with an exception being the dose to the ipsilateral hippocampus head which was similar between protons and photons (Figure 2, Table 2, Figure A2).

**FIGURE 2:**
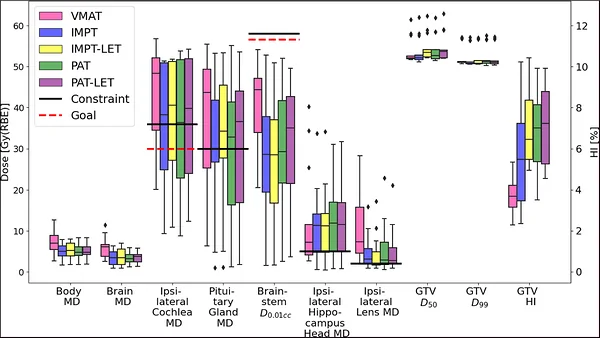
Selected DVH metrics for all modalities. Dose is physical dose for VMAT given in Gy and RBE1.1‐weighted dose for proton modalities given in Gy(RBE). Goals (red) and constraints (black) taken from the clinical goals are shown as horizontal lines for the parameters with relevant associated clinical goals and constraints. MD: mean dose, HI: Homogeneity Index.

**TABLE 2:** Dose statistics for the GTV and OARs for the median patient and range with differences for the VMAT, IMPT and PAT plans.

Dose Statistics	VMAT	IMPT	PAT	VMAT—IMPT	VMAT—PAT	IMPT—PAT
Body MD [Gy(RBE)]	6.98 [2.62, 12.67]	5.03 [1.71, 7.90]	4.71 [1.77, 8.36]	2.03 [0.31, 5.54]^***^	2.08 [0.51, 4.62]^***^	−0.19 [−0.92, 1.38]
Brain MD [Gy(RBE)]	6.04 [2.54, 11.44]	3.44 [0.90, 6.31]	3.16 [1.18, 5.91]	1.98 [−1.81, 5.99]^**^	2.01 [0.11, 5.76]^***^	−0.09 [−0.81, 2.28]
Ipsilateral Cochlea MD [Gy(RBE)]	48.42 [20.04, 56.84]	38.27 [9.32, 51.38]	36.35 [8.76, 53.84]	5.79 [−2.74, 18.61]^**^	4.94 [−0.49, 19.66]^**^	1.00 [−2.97, 3.61]
Pituitary Gland MD [Gy(RBE)]	43.76 [6.31, 55.46]	29.98 [1.01, 53.28]	32.86 [1.21, 55.09]	5.30 [−3.35, 32.19]^**^	8.95 [−1.15, 28.11]^**^	0.50 [−6.41, 10.92]
Brainstem *D*_0.01cc_ [Gy(RBE)]	44.42 [20.49, 54.18]	28.61 [1.56, 52.76]	29.33 [2.59, 52.01]	12.71 [1.42, 29.57]^***^	7.67 [1.66, 22.96]^***^	−0.96 [−12.51, 7.63]
Ipsilateral Hippocampus Head MD [Gy(RBE)]	7.16 [2.67, 40.25]	11.30 [0.57, 33.71]	11.89 [0.76, 31.01]	−0.49 [−14.94, 28.27]	−0.06 [−17.21, 19.29]	−0.02 [−8.98, 10.06]
Ipsilateral Lens MD [Gy(RBE)]	7.32 [1.89, 28.31]	3.05 [0.86, 15.86]	2.93 [0.60, 22.83]	5.39 [−1.53, 24.91]^**^	5.47 [−8.50, 24.71]^**^	−0.35 [−6.97, 1.79]
GTV *D*_50_ [Gy(RBE)]	52.12 [51.73, 61.49]	52.14 [51.20, 62.01]	52.74 [51.44, 61.94]	0.18 [−0.90, 0.98]	−0.63 [−1.66, 0.71]^*^	−0.39 [−1.55, 0.26]^**^
GTV *D*_99_ [Gy(RBE)]	51.10 [50.65, 57.10]	50.84 [50.58, 57.19]	51.23 [50.32, 57.64]	0.29 [−0.41, 0.71]	−0.04 [−0.70, 0.83]	−0.23 [−0.73, 0.42]
GTV HI [%]	3.68 [2.27, 5.36]	5.48 [2.36, 10.23]	7.01 [3.50, 9.93]	−1.61 [−5.96, 1.12]^*^	−3.18 [−5.65, 0.41]^**^	−1.33 [−3.01, 1.50]^*^

*p < 0.05

**p < 0.01

***p < 0.001 from Wilcoxon signed rank test. MD: Mean Dose, HI: Homogeneity Index.

**TABLE 3:** Robustness of target coverage for the IMPT, LET‐optimized IMPT, PAT and LET‐optimized PAT plan. Pass rates for D95% > 95% considering 45 scenarios are provided for the CTV for the initial plan and boost plan, and for the GTV only for the boost plan. Worst case scenarios are also given. The provided values are average values and ranges.

IMPT		IMPT‐LET		PAT		PAT‐LET	
Pass Rate [%]	Worst case scenario [Gy(RBE)]	Pass Rate [%]	Worst case scenario [Gy(RBE)]	Pass Rate [%]	Worst case scenario [Gy(RBE)]	Pass Rate [%]	Worst case scenario [Gy(RBE)]
100 [100, 100]	35,7 [35.23, 36.06]	92,15 [67, 100]	34,0 [30.98, 35.54]	100 [98, 100]	35,1 [34.08, 35.74]	93 [67, 100]	34,0 [32.41, 35.32]
100 [100, 100]	35,8 [35.18, 36.18]	91 [67, 100]	33,0 [25.13, 35.57]	100 [100, 100]	35,3 [34.24, 35.92]	92 [67, 100]	33,9 [31.95, 35.34]
100 [100, 100]	15,8 [13.9, 23.13]	94 [76, 100]	15,1 [12.84, 23.16]	100 [98, 100]	15,7 [13.57, 23.23]	92 [67, 100]	15,1 [12.42, 23.07]

LET optimization showed a slight to moderate statistically significant decrease in LET in the brainstem, pituitary gland and ipsilateral cochlea and a statistically significant increase in LET in the GTV for both IMPT and PAT (Figure 3, Tables A5, A6, A9, A10). Mean LET in the GTV went from 2.5 keV/µm [2.1, 2.8] to 3.4 keV/µm [2.7, 4.1] for IMPT and from 2.8 keV/µm [2.5, 3.1] to 3.4 keV/µm [2.7, 4.0] for PAT for the initial plan (Figure 3, Table A5). The LET in LET optimized OARs was statistically significantly lowered by up to 1 keV/µm while similar LET was maintained in the larger surrounding structures, namely the brain and body (Figure 3, Figure A2, Tables A5, A6). Of the investigated LET metrics and structures, the lowest LET was achieved with IMPT over PAT, but there were considerable differences between the structures, metrics and between the initial plans and the boost plans (Figure 3, Tables A5, A6).

**FIGURE 3:**
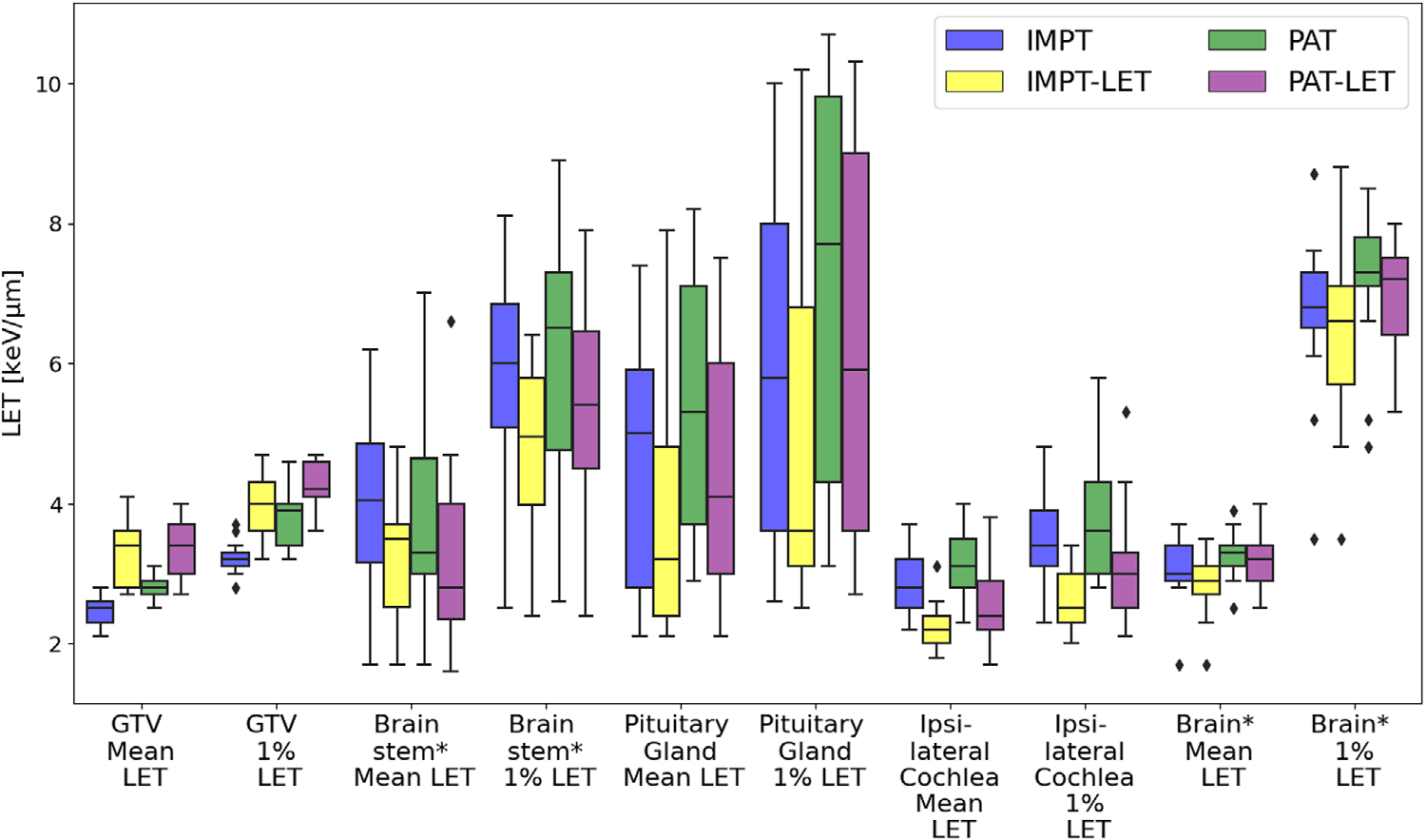
Selected LET‐VH metrics for all proton modalities. All metrics are for the initial plan disregarding the boost plan. Mean LET and the highest LET values (LET to 1% of the organ volume) are shown for (from left to right) the GTV, the brainstem, the pituitary gland, the ipsilateral cochlea and the brain. *The brainstem and brain have a dose cutoff where the LET‐VH parameter considers only the fraction of the organ volume receiving dose over 15 Gy(RBE).

While RBE_1.1_‐weighted GTV dose was similar between proton plans, the RBE_VAR_‐weighted doses were statistically significantly higher for the LET‐optimized plans compared to the non‐LET optimized plans (Figure 4, Tables A7, A8, A11–A13). Percentage increase in median GTV dose from RBE_1.1_‐weighted dose to RBE_LYN_‐weighted dose was 12.6% [10.3%, 14.6%] for the non‐LET optimized IMPT plan, 17.5% [13.5%, 21.6%] for the LET‐optimized IMPT plan, 14.1% [12.5%, 15.8%] for the non‐LET optimized PAT plan and 17.8% [13.2%, 21.4%] for the LET‐optimized PAT plan. RBE_LYN_‐weighted doses in OARs were also slightly reduced with both modalities for some structures such as the ipsilateral cochlea, brainstem and ipsilateral hippocampus head, but none of the differences were statistically significant (Figure 4, Table A7).

**FIGURE 4:**
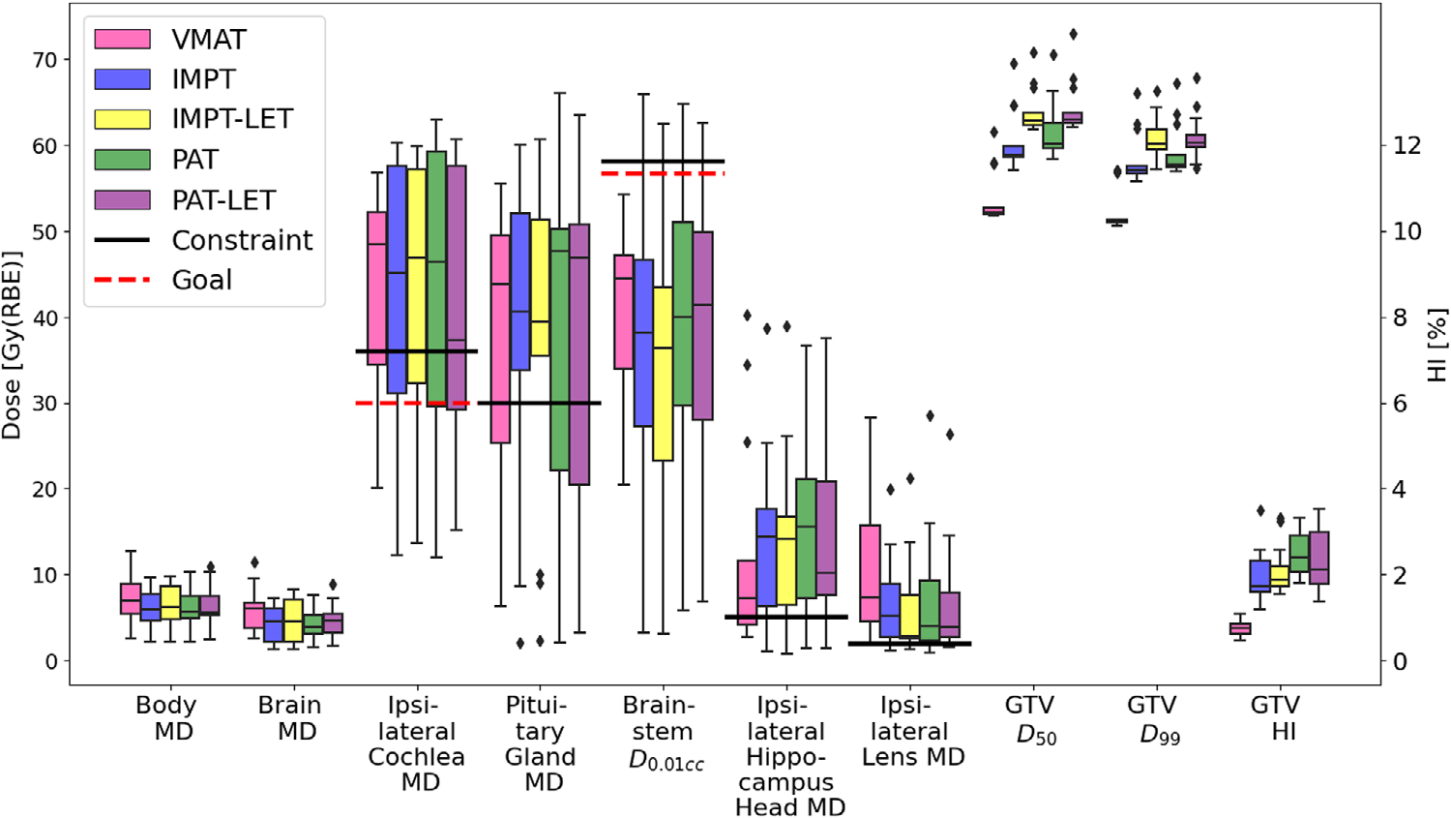
Selected DVH metrics for all modalities with RBE‐weighted dose from the Lyngholm model compared to physical dose for VMAT. Goals (red) and constraints (black) taken from the clinical goals are shown as horizontal lines for the parameters with relevant associated clinical goals and constraints. MD: mean dose, HI: Homogeneity Index.

Median delivery time was 134.5 [95.3, 203.7] seconds for the initial IMPT plan and 197.4 [138.9, 244.6] seconds for the initial PAT plan. LET optimization reduced the delivery times slightly to 133.9 [92.0, 201.7] seconds for the initial IMPT plan and 192.3 [134.7, 229.6] seconds for the initial PAT plan (Table 3, Figure 5). The boost plans had similar delivery times for PAT but shorter delivery times for IMPT, increasing the gap between the two modalities (Table 4, Figure A3). The number of energy layers and the number of spots were both higher for PAT than for IMPT, and LET optimization resulted in a reduction in the number of spots, while energy layer number remained similar (Table 4).

**FIGURE 5:**
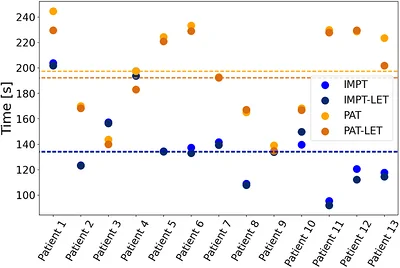
Delivery times for the initial plan for IMPT, LET‐optimized IMPT, PAT and LET‐optimized PAT for all patients. The median patient is given by the dashed lines.

**TABLE 4:** Beam delivery time [s], number of energy layers and number of spots for the intial plan and boost plan for IMPT, LET‐optimized IMPT, PAT and LET‐optimized PAT. Symbols after IMPT‐LET and PAT‐LET refer to the significance of the differences from before and after LET optimization. Median values and range are provided.

Initial Plan					
	IMPT	IMPT‐LET	PAT	PAT‐LET	Difference IMPT—PAT
Beam Delivery Time [s]	134.5 [95.3, 203.7]	133.9 [92.0, 201.7]^*^	197.4 [138.9, 244.6]	192.3 [134.7, 229.6]^**^	−62.9 ^***^
Energy Layers [#]	87 [66, 138]	84 [64, 134]	130 [85, 157]	130 [85, 157]	−43 ^**^
Number of Spots [#]	4749 [2281, 12574]	4534 [1769, 10252]^*^	10265 [1269, 30311]	6703 [1352, 27425]^**^	−5516 ^**^

Boost Plan					
	IMPT	IMPT‐LET	PAT	PAT‐LET	Difference IMPT—PAT
Beam Delivery Time [s]	104.8 [41.1, 226.5]	104.3 [38.9, 222.0]^***^	192.5 [115.9, 247.4]	181.4 [113.2, 247.3]^***^	−80.2 ^***^
Energy Layers [#]	71 [33, 159]	71 [32, 159]	130 [68, 159]	107 [68, 145]	−59 ^***^
Number of Spots [#]	2474 [1124, 8362]	1611 [671, 7717]^***^	4647 [1252, 16258]	2672 [401, 14069]^**^	−2173^***^

*p < 0.05

**p < 0.01

***p < 0.001 from Wilcoxon signed rank test.

## 4 DISCUSSION

In this study, VMAT, IMPT, and PAT plans were evaluated for pediatric PM‐RMS patients, with an additional focus on LET optimization for the proton modalities. PAT resulted in similar dose to OARs compared with IMPT, but with 1–2 minute longer delivery times and increased plan complexity due to a higher number of spots and energy layers. LET optimization increased LET in the GTV while maintaining similar dose to the OARs, leading to the intended increase in variable RBE‐weighted dose deposited in gross disease. Delivery times were also reduced by a few seconds with LET optimization due to a reduction in the number of spots used by the plans. The slightly increased physical dose in the OARs was mitigated by reduced LET_,_ which subsequently resulted in little change in variable RBE‐weighted dose. All proton planning strategies showed superior dose distributions compared to VMAT.

Given high rates of local failure^2^, ^4^ and significant toxicity,^1^, ^3^ PM‐RMS patients should be ideal candidates for LET optimization, where our results indicated similar potential for both IMPT and PAT. Our results demonstrated the potential of LET optimization to enhance the biological effectiveness within the tumor while protecting critical OARs around the skull base in this radiosensitive population. Comparisons between passively scattered protons and intensity modulated radiotherapy using photons for the patient group have shown similar target coverage but improved OAR sparing with protons^7^, ^10^ which was confirmed by our results. While the magnitude of improvements from IMPT to PAT with and without LET optimization varied considerably across patients, there is a clear potential for benefit, and these findings open the door for ongoing research to (a) further refine these new treatment techniques and (b) identify the patients who would derive the greatest therapeutic ratio.

The clinical significance of redistributing high LET values between OARs and target volumes is, however, uncertain. Relatively few studies have focused on LET and recurrence, but Sethi. et al^33^ did not find LET in the target to be associated with recurrence in a pediatric medulloblastoma cohort, which suggests the 0.5‐1.0 keV/µm increase in LET in the target observed in this study to be of limited clinical significance. There are more studies investigating LET and toxicities, particularly image changes on MRI and more severe necrosis. Of the organs considered in this manuscript, the brainstem is likely the most associated with LET‐related toxicities. Bregman et al.^34^ found image changes post proton therapy to be associated with elevated LET and to be most prevalent in the pons substructure of the brainstem. Thus, the 0.6 keV/µm reduction in 1% LET seen in this work for the PAT plan could potentially be associated with a decreased risk of radiation‐induced image changes, although further similar studies are required to strengthen the clinical evidence.

LET optimization was effective for both IMPT and PAT plans in this study with neither modality emerging as clearly superior. This differs slightly from what is reported by Li et al.^24^ and Bertolet et al.^23^ who both found PAT to improve the potential of LET optimization compared to standard IMPT. This is likely explained by the use of dynamic arcs in our study and the beams’ dependency on adjacent beams’ energy layers. Compared to discrete PAT, dynamic PAT introduces a considerable limitation on the degrees of freedom available for adjusting LET values. There are few studies directly comparing dynamic and discrete proton arc therapy on clinical cohorts, but Wuyckens demonstrates in chapter 2.4.2. of her PhD thesis how energy layers of dynamic arcs, unlike discrete arcs, are considerably more dependent on the energy layers of adjacent arcs and are limited to one energy layers per angles.^35^

When LET optimization was not considered, there was slightly increased maximum LET with PAT, similarly to the findings of Reiners et al.^36^ for adult head and neck cancer patients. Toussaint et al.,^15^ on the other hand, found slightly decreased maximum LET in the brainstem with PAT compared to IMPT for four pediatric brain tumors, and Cong et al.^37^ similarly saw a slight decrease in both maximum and mean LET with PAT for ten adult esophageal cancer patients. Cong et al. further found a slight, but statistically insignificant, increase in mean LET in the CTV with PAT compared to IMPT which is also what we observed in this study.

LET optimization has seen an increase in research focus in later years and has also been explored in other pediatric cohorts. Goudarzi et al. directly compared LET optimization and variable RBE optimization and their ability to decrease LET and variable RBE in OARs and increase LET and variable RBE in the CTV in pediatric ependymoma patients.^38^ They found LET optimization to consistently increase mean LET in the target by around 0.1‐1.3 keV/µm, similarly to our findings, while both LET optimization and variable RBE optimization lowered both LET and variable RBE in OARs. Torsha et al.^39^ similarly found increased LET in the target and mostly decreased LET in OARs with LET optimization compared to robust optimization for three pediatric ependymoma patients.

Dose distributions were fairly similar across all proton modalities but trending towards lower OAR doses with PAT. This is consistent with previous studies showing improved OAR sparing with proton arcs over standard IMPT, using both dynamic arc and static arc techniques, although the impact on delivery time remains uncertain.^40^, ^41^, ^42^ In this study, however, some exceptions were observed, most notably an increase in maximum brainstem dose with PAT compared to IMPT. This can likely be attributed to the size and position of the organ relative to the target volume, allowing for easier avoidance with discrete beams compared to continuous arcs. For IMPT we also found decreased brainstem dose using LET optimization. This is likely explained by the displacement of spots away from the volume, to ensure the distal fall‐off is shifted to surrounding tissue. The dose values in the brainstem were too low to be weighted highly in a dose‐only optimization, but the LET‐optimization more highly prioritized the removal of these spots, which incidentally resulted in a further dose‐reduction.

An alternative to LET optimization is surrogates for LET which avoid the problem of needing to pinpoint exact LET thresholds based on limited clinical evidence. Variable RBE‐based optimization has been explored in several studies^38^, ^43^, ^44^ and targets a parameter that can be directly associated with both LET and dose. Other options include track‐end optimization where the number of particle track‐ends, which are known to be associated with elevated LET values, are restricted within OARs and potentially placed in target volumes instead,^44^ and ‘dirty dose’ where dose with high LET is restricted to a greater degree than dose with low LET.^44^ Additionally, “beam pruning” of PAT plans has been suggested as an option where high energy layers are selectively removed from the proton arc in order to increase the LET in the target and lower the LET in OARs.^45^

The presentation of LET results is an additional aspect warranting discussion given the limited interpretive value of LET without a corresponding dose parameter. Consequently, methods for reporting and accounting for LET in clinical practice remains a debated topic with different centers adopting varying strategies.^46^, ^47^ A variable RBE model accurately describing the effect of protons on human tissue would be the ideal choice for reporting LET in conjunction with dose. However, since RBE in cell cultures and human tissue are based on different endpoints, there is no guarantee for the clinical applicability of these variable RBE models. Additionally, α/β values add another aspect of uncertainty for the variable RBE models, where an overestimation of the α/β value of a tissue could underestimate the impact of LET on the variable RBE‐weighted doses.^29^ Thus, we chose to report LET above certain dose thresholds in addition to model‐based variable RBE, acknowledging that LET volume metrics above dose thresholds do not pinpoint the positions where both dose and LET are at their peak.

Beam configuration has also been shown to have a considerable impact on resulting LET distributions^43^, ^48^, ^49^ which could have impacted the results presented in this work. The combination of heterogeneously positioned tumors, several organs at risk limiting available entry points, and the focus on PAT made the exploration of angle dependency secondary in this work. LET optimization may also have a particular benefit for IMPT since the impact of entry angles may be less critical through the use of LET optimization, thus allowing improved flexibility in beam angles stopping closer to OARs.

The impact of LET optimization on plan robustness is an important consideration and our results indicate a slight loss in robustness after LET optimization. This is in line with prior studies on both proton and carbon ion therapy that have demonstrated that increasing the LET in the target volume can conflict with dose uniformity and plan robustness, particularly an increase in range uncertainty.^50^, ^51^, ^52^ Comparisons between IMPT and PAT have shown improved robustness with PAT,^53^, ^54^ which could help offset the loss of robustness associated with LET optimization. Additionally, the impact of range uncertainty has been shown to be less pronounced in PAT than in IMPT.^55^

## 5 CONCLUSION

In conclusion, PAT and IMPT demonstrated comparable dose advantage over VMAT in this study. LET optimization enabled more favorable LET distributions with both IMPT and PAT, with minimal detrimental effect on surrounding healthy tissue and OARs not specifically considered in the LET optimization. However, specific LET thresholds and relative weighting with dose criteria varied considerably based on tumor location, emphasizing the need for larger cohorts of anatomically similar patients to develop concrete, patient‐specific guidelines for LET optimization.

## AUTHOR CONTRIBUTIONS

**Andreas Havsgård Handeland**: Conceptualization; data curation; formal analysis; methodology; visualization; writing—original draft. **Hardev Singh Grewal**: Conceptualization; data curation; methodology; writing—review and editing. **Camilla Grindeland Boer**: Data curation; methodology; writing—review and editing. **Daniel J. Indelicato**: Conceptualization; methodology; writing—review and editing. **Kristian Smeland Ytre‐Hauge**: Conceptualization; methodology; writing—review and editing. **Helge Henjum**: Conceptualization; methodology; writing—review and editing. **Camilla Hanquist Stokkevåg**: Conceptualization; methodology; supervision; writing—review and editing

## CONFLICT OF INTEREST STATEMENT

The authors declare no conflicts of interest.

## ETHICS STATEMENT

The use of the patients was approved by the institutional review board at UFHPTI and the Norwegian regional ethics committee (REK602921).

## Supporting information



Supporting Information

Supporting Information

## Data Availability

Data is available upon reasonable request to the corresponding author and approval by the Institution's Review Board.
