# Supplementary material for: Dynamic Proton arc therapy for paediatric parameningeal rhabdomyosarcoma
Source: J Appl Clin Med Phys. 2026 Oct 20;27(11):e70867. doi: 10.1002/acm2.70867 (PMC13682623; doi:10.1002/acm2.70867)
Supplement: Supplementary file 1 [file ACM2-27-e70867-s001.pdf]

Supplementary

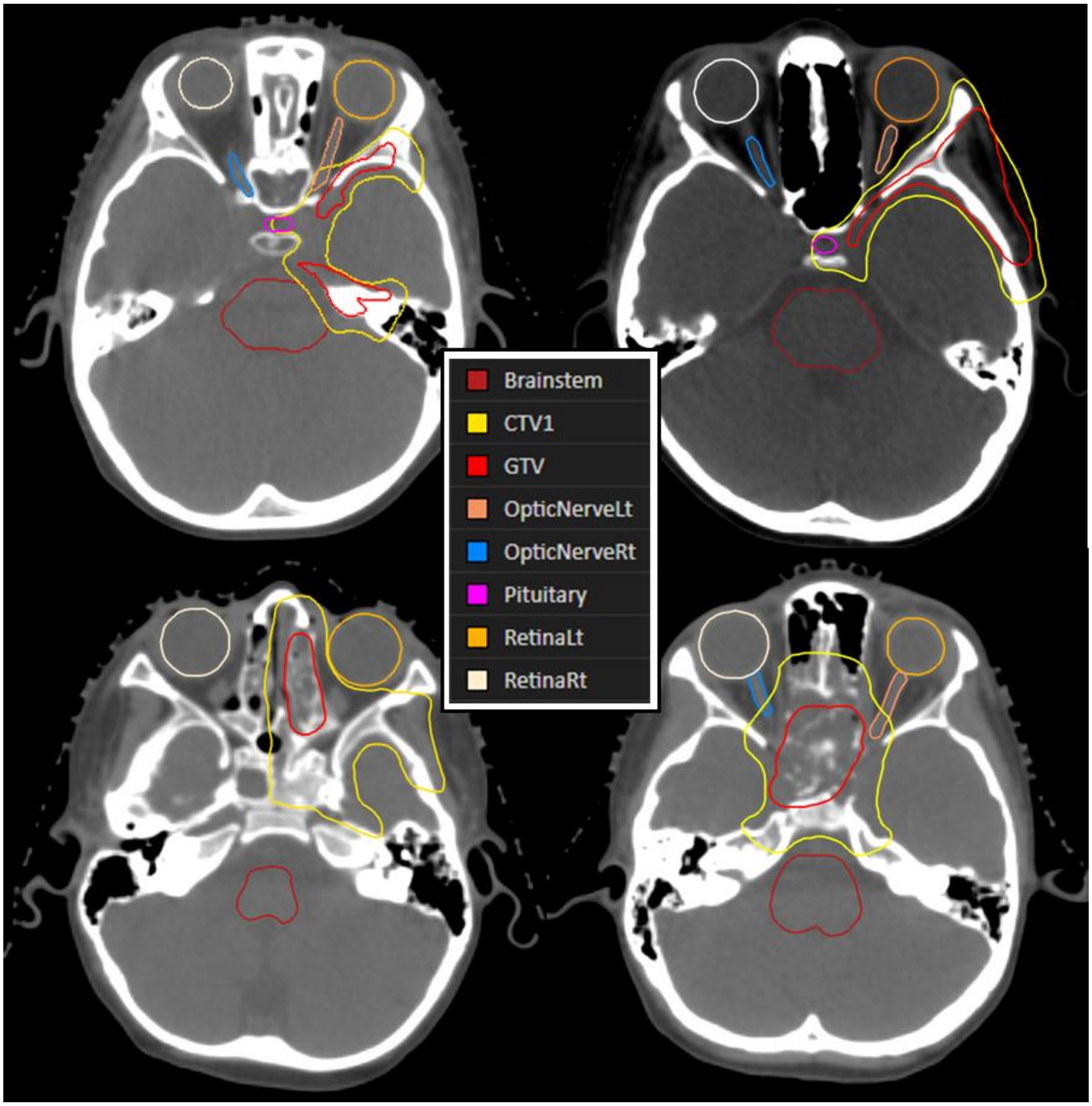

Figure A1: Tumor locations and adjacent OARs for four example patients.

Homogeneity Index

$$HI = \frac{D_2 - D_{98}}{D_p} \cdot 100\% \tag{A1}$$

Here D_2 and D_{98} are doses at the specified volume points, approaching maximum and minimum dose, respectively, while D_p is prescribed dose. A low percentage correlates to good homogeneity within the target volume.

Variable RBE models

Both the Lyngholm RBE model and the McNamara RBE model are based on the linear-quadratic model formulated as follows (A2):

$$\mathrm{RBE}\left(D_p, \left(\frac{\alpha}{\beta}\right)_x, \mathrm{RBE}_{\max}, \mathrm{RBE}_{\min}\right) = \frac{1}{2D_p}\left(\sqrt{\left(\frac{\alpha}{\beta}\right)_x^2 + 4D_P\left(\frac{\alpha}{\beta}\right)_x \mathrm{RBE}_{\max} + 4D_p^2\mathrm{RBE}_{\min}^2} - \left(\frac{\alpha}{\beta}\right)_x\right) \quad \text{(A2)}$$

$\mathrm{RBE}_{\min}$ for the Lyngholm RBE model is given by (A3):

$$\mathrm{RBE}_{\min} = 1.021 + 0.0268\left(\frac{\mathrm{keV}}{\mu\mathrm{m}}\right)^{-1} \cdot \mathrm{LET_d} \quad \text{(A3)}$$

$\mathrm{RBE}_{\max}$ for the Lyngholm RBE model is given by (A4):

$$\mathrm{RBE}_{\max} = 1.161 + \frac{0.328\left(\frac{\mathrm{keV}}{\mu\mathrm{m}}\right)^{-1}}{\left(\frac{\alpha}{\beta}\right)_x} \cdot \mathrm{LET_d} \quad \text{(A4)}$$

$\mathrm{RBE}_{\min}$ for the McNamara model is given by (A5):

$$\mathrm{RBE}_{\min} = 1.1012 - 0.0038703\sqrt{\left(\frac{\alpha}{\beta}\right)_x}\left(\frac{\mathrm{keV}}{\mu\mathrm{m}}\right)^{-1} \cdot \mathrm{LET_d} \quad \text{(A5)}$$

RBE_{max} for the McNamara RBE model is given by (A6):

$$\mathrm{RBE_{max}} = 0.99064 + \frac{0.35605\left(\frac{\mathrm{keV}}{\mu\mathrm{m}}\right)^{-1}}{\left(\frac{\alpha}{\beta}\right)_x} \cdot \mathrm{LET_d} \tag{A6}$$

The LET-weighted dose, or Unkelbach model, describes RBE as a linear function with LET, as follows (A7):

$$\mathrm{RBE} = 1 + \kappa \cdot \mathrm{LET_d} \tag{A7}$$

Where κ is a fitting parameter where a value 0.04 was used as initially proposed by Unkelbach et al.

Table A1: Patient and plan characteristics.

Patient no.	Prescribed Dose [Gy(RBE)]	Skull Base Laterality
1	36.0 + 14.4	Left
2	36.0 + 14.4	Right
3	36.0 + 14.4	Right
4	36.0 + 14.4	Left
5	36.0 + 14.4	Left
6	36.0 + 14.4	Central
7	36.0 + 14.4	Right
8	36.0 + 23.4	Right
9	36.0 + 19.8	Left
10	36.0 + 19.8	Left
11	36.0 + 14.4	Left
12	36.0 + 14.4	Right
13	36.0 + 14.4	Left

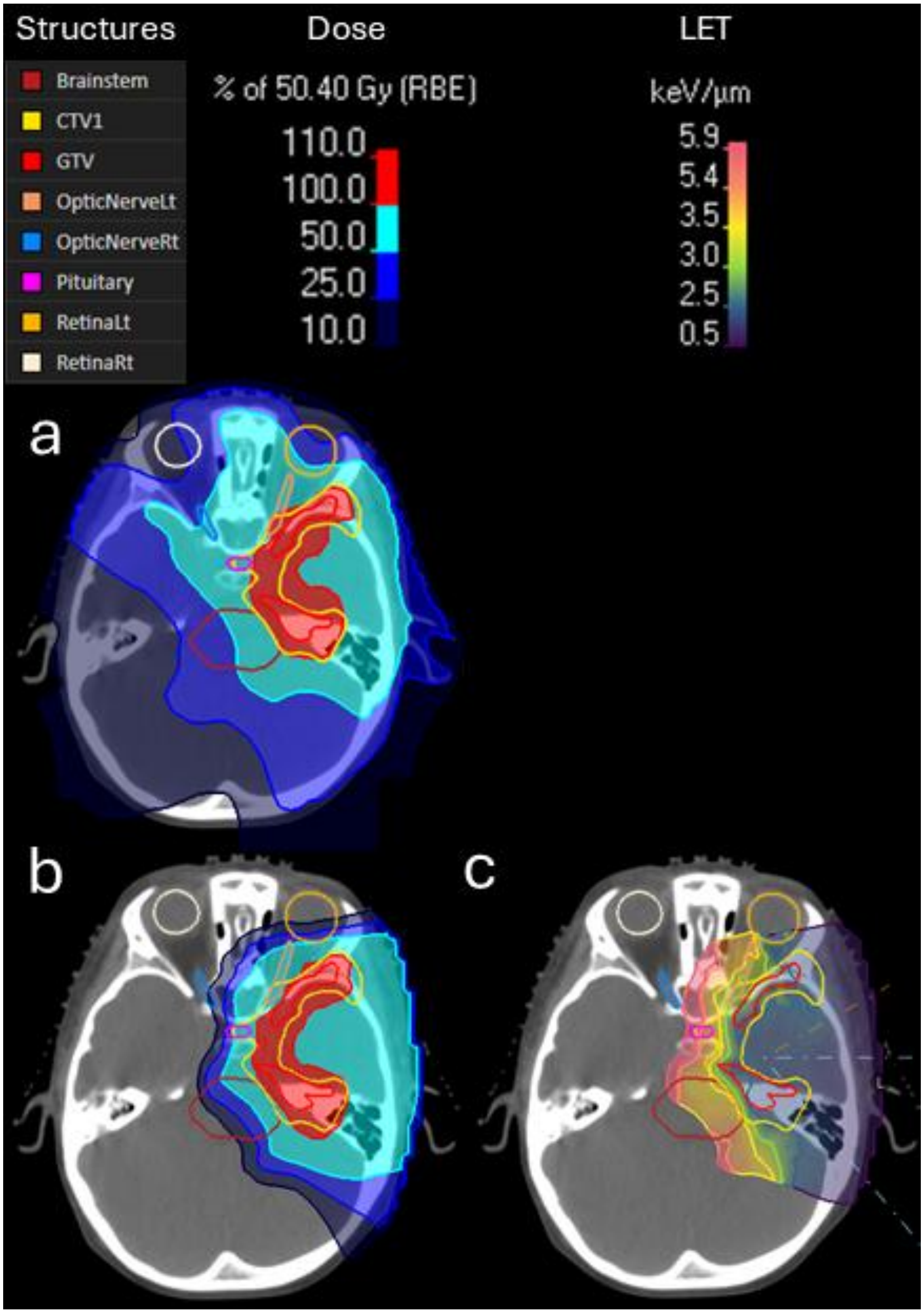

Structures
Dose
LET
Brainstem
CTV1
GTV
OpticNerveLt
OpticNerveRt
Pituitary
RetinaLt
RetinaRt
% of 50.40 Gy (RBE)
110.0
100.0
50.0
25.0
10.0
keV/µm
5.9
5.4
3.5
3.0
2.5
0.5
a
b
c

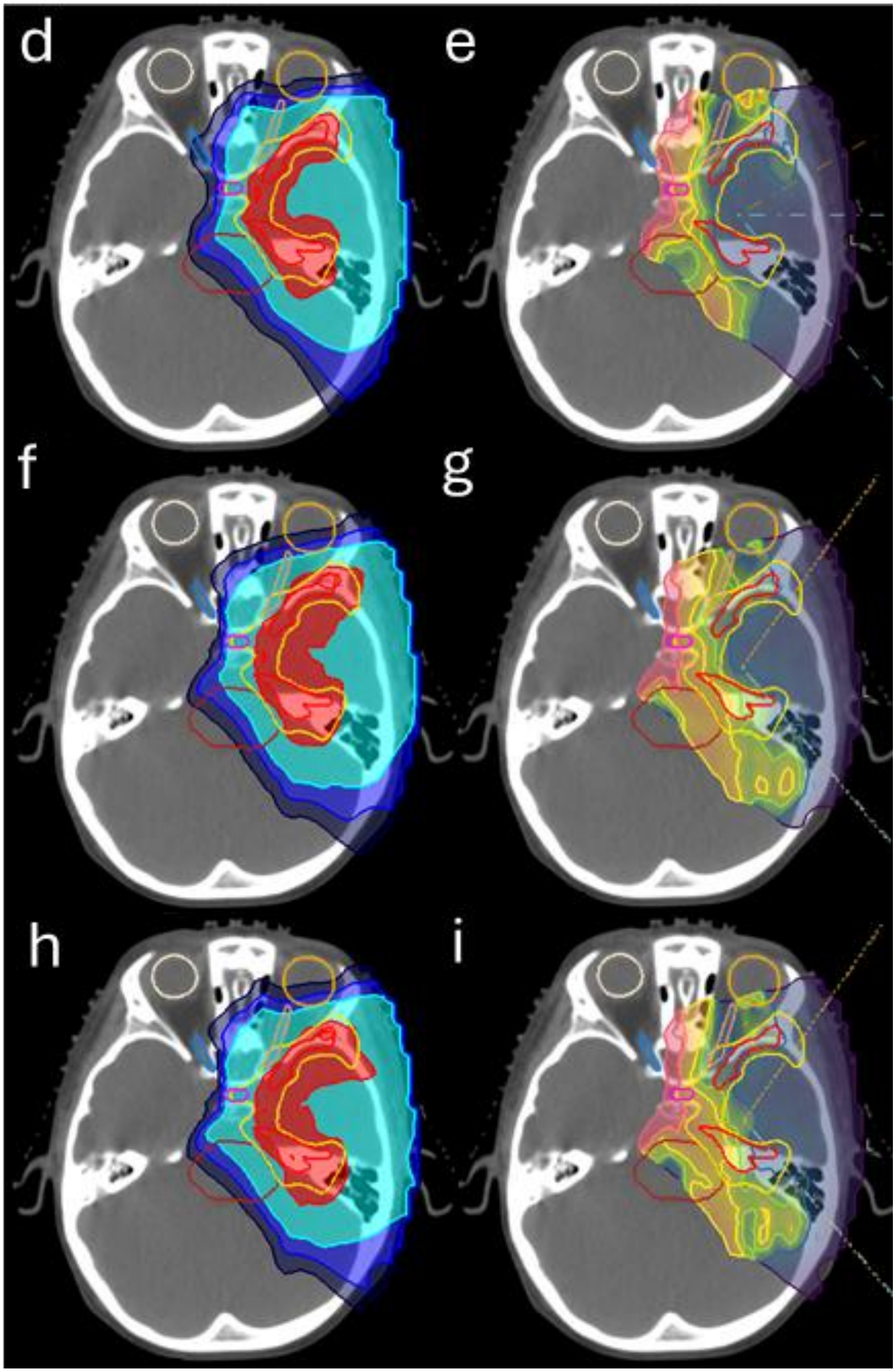

Figure A2: Dose and LET distributions for one example patient including all modalities. a) Dose-distribution VMAT, b) Dose-distribution IMPT, c) LET-distribution IMPT, d) dose-distribution LET-optimized IMPT, e) LET-distribution LET-optimized IMPT, f) dose-distribution PAT, g) LET-distribution PAT, h) dose-distribution LET-optimized PAT, i) LET-distribution LET-optimized PAT.

Table A2: Average $RBE_{1.1}$-weighted dose statistics for the target and OARs between the two dose planners, including the difference between the two planners, for the IMPT plan and the LET-optimized IMPT plan.

Dose Statistics	IMPT Planner 1	IMPT Planner 2	Difference	IMPT-LET Planner 1	IMPT-LET Planner 2	Difference
CTV D50	54,3 ±4,4	51,2 ±0,3	3,0	54,7 ±4,4	51,9 ±0,5	2,8
CTV D99	42,8 ±4,7	44,2 ±2,7	-1,5	42,6 ±4,6	43,7 ±2,0	-1,1
GTV D50	55,4 ±3,9	51,6 ±0,2	3,8	56,3 ±3,5	52,4 ±0,3	4,0
GTV D99	53,4 ±3,0	50,7 ±0,1	2,7	53,6 ±3,0	50,7 ±0,1	3,0
GTV HI [%]	7,7 ±1,9	3,3 ±0,6	0,0	8,0 ±1,8	5,9 ±1,3	2,1
Body	5,6 ±1,3	4,1 ±1,6	1,6	5,7 ±1,2	4,3 ±1,9	1,4
Brain	3,9 ±1,9	2,6 ±1,8	1,3	4,0 ±1,8	3,1 ±2,3	0,9
Brainstem D10	16,0 ±12,1	12,0 ±13,4	4,0	15,4 ±11,3	12,3 ±13,8	3,2
Brainstem D50	5,1 ±5,1	4,7 ±6,9	0,4	4,5 ±4,3	4,3 ±6,4	0,2
Brainstem D0.01cc	28,4 ±16,3	22,2 ±17,3	6,2	27,8 ±16,5	23,0 ±17,7	4,8
brainstemCore	20,0 ±14,0	15,2 ±13,6	4,8	19,5 ±14,0	15,7 ±14,4	3,8
Cochlea Left	38,8 ±13,8	32,2 ±15,4	6,6	39,5 ±13,4	33,9 ±16,4	5,6
Cochlea Right	5,0 ±8,7	10,0 ±15,8	-5,1	4,9 ±8,9	11,4 ±17,7	-6,5
HippoHead Lt	11,4 ±11,4	11,1 ±7,6	0,3	11,4 ±11,6	12,3 ±8,4	-0,9
HippoHead Rt	1,6 ±1,8	1,0 ±1,9	0,6	1,8 ±1,9	1,4 ±2,1	0,4
HippoTail Lt	3,5 ±5,0	1,5 ±1,4	2,0	3,5 ±4,9	1,7 ±1,9	1,8
HippoTail Rt	0,1 ±0,1	0,2 ±0,3	-0,1	0,1 ±0,1	0,3 ±0,4	-0,2
Hypothalamus	3,2 ±2,0	2,3 ±4,0	1,0	3,4 ±2,3	3,0 ±5,7	0,5
Lacrimal Left	15,9 ±11,0	15,3 ±11,7	0,6	16,1 ±11,9	15,5 ±11,0	0,6
Lacrimal Right	1,1 ±2,2	0,5 ±1,1	0,6	1,2 ±2,5	0,8 ±1,7	0,4

Lens Left	3,5 ±3,5	5,7 ±5,7	-2,2	3,5 ±3,6	5,6 ±6,2	-2,0
Lens Right	0,6 ±0,8	0,4 ±0,3	0,2	0,6 ±0,7	0,4 ±0,3	0,2
Optic Chiasm	14,5 ±13,5	13,3 ±12,8	1,2	14,8 ±13,6	16,2 ±13,1	-1,4
Optic Nerve Lt	42,9 ±9,6	36,8 ±14,6	6,1	42,8 ±10,1	38,9 ±14,2	3,9
Optic Nerve Rt	21,0 ±18,9	15,6 ±12,9	5,4	21,6 ±18,5	20,4 ±15,9	1,2
Pituitary	33,6 ±17,2	23,7 ±17,0	9,9	33,7 ±17,0	26,1 ±18,6	7,6
Retina Left	32,1 ±9,4	32,6 ±16,0	-0,5	32,0 ±9,3	33,2 ±14,6	-1,2
Retina Right	7,3 ±11,5	5,4 ±4,6	1,9	7,4 ±11,9	5,0 ±4,9	2,4
Spinal Cord	4,8 ±3,0	7,4 ±9,2	-2,6	4,3 ±2,8	7,2 ±9,1	-2,9
Left Temp Lobe	36,5 ±21,4	29,2 ±9,6	7,3	36,7 ±20,9	32,7 ±11,2	3,9
Right Temp Lobe	2,9 ±5,1	3,4 ±7,4	-0,5	3,1 ±5,2	3,9 ±7,2	-0,8

Table A3: Average $RBE_{1.1}$-weighted dose statistics for the target and OARs between the two dose planners, including the difference between the two planners, for the PAT plan and the LET-optimized PAT plan.

Dose Statistics	PAT Planner 1	PAT Planner 2	Differen ce	PAT-LET Planner 1	PAT-LET Planner 2	Differen ce
CTV D50	54,7 ±4,4	51,6 ±0,9	3,1	54,7 ±4,4	51,6 ±0,9	3,1
CTV D99	43,0 ±4,8	43,1 ±2,8	-0,1	43,0 ±4,7	44,2 ±2,5	-1,3
GTV D50	55,9 ±3,7	52,1 ±0,7	3,8	56,6 ±3,7	52,3 ±0,3	4,4
GTV D99	53,7 ±3,1	50,9 ±0,4	2,8	53,7 ±3,1	50,7 ±0,2	3,0
GTV HI	8,4 ±0,9	4,8 ±1,2	3,6	8,5 ±1,0	5,4 ±0,9	3,1
Body	6,1 ±1,6	6,1 ±1,6	0,0	3,8 ±1,2	4,1 ±1,2	-0,3
Brain	3,9 ±1,3	3,8 ±1,2	0,1	2,3 ±1,2	2,7 ±1,4	-0,4
Brainstem D10	21,6 ±11,2	20,8 ±11,9	0,8	11,0 ±10,9	13,5 ±12,4	-2,5
Brainstem D50	7,5 ±5,0	6,8 ±5,4	0,7	3,6 ±5,8	5,6 ±8,2	-2,0

Brainstem D0.01cc	34,6<br>±13,6	33,2<br>±14,2	1,3	21,5<br>±15,2	24,2<br>±16,6	-2,8
brainstemCore	27,2<br>±12,5	26,4<br>±13,3	0,8	14,2<br>±11,5	17,2<br>±13,7	-3,0
Cochlea Left	38,5<br>±16,0	38,4<br>±16,0	0,1	31,5<br>±16,8	33,5<br>±15,7	-1,9
Cochlea Right	4,9<br>±11,0	4,8<br>±10,9	0,0	8,7<br>±13,7	9,3<br>±14,6	-0,6
HippoHead Lt	13,2<br>±10,7	13,0<br>±10,8	0,2	9,3<br>±7,8	9,6<br>±7,4	-0,3
HippoHead Rt	1,1<br>±1,8	1,0<br>±1,8	0,0	0,8<br>±1,8	1,4<br>±3,3	-0,6
HippoTail Lt	3,8<br>±4,7	3,5<br>±4,6	0,3	1,2<br>±1,0	1,4<br>±1,2	-0,2
HippoTail Rt	0,1<br>±0,1	0,1<br>±0,1	0,0	0,1<br>±0,2	0,3<br>±0,7	-0,2
Hypothalamus	3,2<br>±1,9	3,4<br>±2,0	-0,2	0,7<br>±0,7	0,8<br>±1,0	-0,1
Lacrimal Left	19,3<br>±15,5	19,1<br>±15,6	0,2	14,0<br>±12,8	15,8<br>±13,0	-1,9
Lacrimal Right	0,7<br>±1,3	0,7<br>±1,3	0,0	0,9<br>±2,1	0,7<br>±1,5	0,2
Lens Left	4,1<br>±4,4	3,8<br>±3,8	0,3	7,0<br>±8,4	7,3<br>±7,7	-0,3
Lens Right	0,7<br>±0,6	0,7<br>±0,6	0,0	0,8<br>±1,1	0,6<br>±0,6	0,2
Optic Chiasm	16,3<br>±13,2	18,0<br>±12,7	-1,6	9,7<br>±11,4	10,2<br>±12,7	-0,5
Optic Nerve Lt	45,4<br>±6,2	46,0<br>±6,0	-0,6	33,2<br>±17,3	33,9<br>±16,9	-0,7
Optic Nerve Rt	21,7<br>±19,3	21,4<br>±19,5	0,4	6,4<br>±10,0	9,3<br>±12,1	-2,9
Pituitary	34,5<br>v16,6	36,0<br>±15,8	-1,5	19,5<br>±16,2	20,8<br>±17,7	-1,3
Retina Left	33,8<br>±8,5	34,0<br>±8,3	-0,2	30,0<br>±16,4	31,5<br>±15,8	-1,6
Retina Right	9,0<br>±12,5	8,3<br>±11,9	0,6	4,7<br>±5,1	3,5<br>±3,0	1,2
Spinal Cord	6,7<br>±3,7	6,5<br>±3,3	0,2	5,7<br>±7,6	5,5<br>±6,2	0,2
Left Temp Lobe	40,2<br>±20,5	39,6<br>±19,0	0,5	26,9<br>±10,6	29,2<br>±11,1	-2,3
Right Temp Lobe	3,2<br>±6,2	3,2<br>±6,2	0,0	2,3<br>±5,3	2,1<br>±4,8	0,2

Table A4: Dose statistics for the GTV and OARs for the median patient and range with differences. Both LET-optimized and non-LET optimized IMPT and PAT plans are shown and the differences between LET and non-LET optimized plans. *p < 0.05, **p < 0.01, ***p < 0.001 from Wilcoxon signed rank test. MD: Mean Dose, HI: Homogeneity Index.

Dose Statistics	IMPT	IMPT-LET	Difference	PAT	PAT-LET	Difference
Body MD [Gy(RBE)]	5.03 [1.71, 7.90]	5.18 [1.75, 7.96]	-0.04 [-1.14, 0]**	4.71 [1.77, 8.36]	4.73 [1.90, 8.35]	-0.02 [-0.63, 0.14]
Brain MD [Gy(RBE)]	3.44 [0.90, 6.31]	3.43 [0.92, 6.93]	-0.02 [-2.05, 0.14]	3.16 [1.18, 5.91]	3.79 [1.27, 5.72]	-0.02 [-0.63, 0.28]
Ipsilateral Cochlea MD [Gy(RBE)]	38.27 [9.32, 51.38]	40.61 [10.91, 51.80]	-0.90 [-5.28, 2.44]*	36.35 [8.76, 53.84]	39.85 [12.29, 54.24]	-0.12 [-5.87, 1.52]
Pituitary Gland MD [Gy(RBE)]	29.98 [1.01, 53.28]	34.29 [1.08, 53.33]	-0.30 [-7.06, 0.72]*	32.86 [1.21, 55.09]	36.57 [1.72, 53.63]	-0.28 [-6.86, 1.46]
Brainstem $D_{0.01cc}$ [Gy(RBE)]	28.61 [1.56, 52.76]	28.51 [1.61, 50.96]	0.03 [-7.72, 8.41]	29.33 [2.59, 52.01]	35.10 [3.63, 49.59]	-0.36 [-6.76, 6.97]
Ipsilateral Hippocampus Head MD [Gy(RBE)]	11.30 [0.57, 33.71]	11.16 [0.46, 34.18]	0 [-6.16, 0.97]	11.89 [0.76, 31.01]	11.52 [0.78, 31.75]	0.09 [-2.35, 1.78]
Ipsilateral Lens MD [Gy(RBE)]	3.05 [0.86, 15.86]	1.94 [0.91, 17.14]	-0.03 [-1.28, 1.38]	2.93 [0.60, 22.83]	2.82 [0.89, 21.07]	-0.11 [-2.96, 1.89]
GTV D_{50} [Gy(RBE)]	52.14 [51.20, 62.01]	53.48 [52.11, 62.33]	-0.92 [-1.46, -0.22]***	52.74 [51.44, 61.94]	53.66 [52.01, 62.94]	-0.50 [-1.29, 1.00] *
GTV D_{99} [Gy(RBE)]	50.84 [50.58, 57.19]	50.82 [50.56, 57.00]	0.03 [-0.72, 0.23]	51.23 [50.32, 57.64]	51.03 [50.35, 57.30]	-0.03 [-0.23, 0.66]
GTV HI [%]	5.48 [2.36, 10.23]	6.45 [4.95, 10.43]	-1.10 [-4.33, 0.79]**	7.01 [3.50, 9.93]	7.23 [4.55, 9.91]	-0.35 [-1.78, 1.24]

Table A5: Initial plan LET statistics for the GTV and OARs for the median patient and range with differences. Both LET-optimized and non-LET optimized IMPT and PAT plans are shown and the differences between LET and non-LET optimized plans. *p < 0.05, **p < 0.01, ***p < 0.001 from Wilcoxon signed rank test.

LET Statistics [keV/µm]	IMPT	IMPT-LET	Difference (IMPT – IMPT-LET)	PAT	PAT-LET	Difference (PAT – PAT-LET)
GTV Mean LET	2.5 [2.1, 2.8]	3.4 [2.7, 4.1]	-0.9 [-1.8, -0.2]***	2.8 [2.5, 3.1]	3.4 [2.7, 4.0]	-0.5 [-1.5, 0.1]***
GTV 1% LET	3.2 [2.8, 3.7]	4 [3.2, 4.7]	-1 [-1.5, 0.3]**	3.9 [3.2, 4.6]	4.2 [3.6, 4.7]	-0.2 [-1.3, 0]**
Brainstem (*D* > 15 Gy) Mean LET	4.1 [1.7, 6.2]	3.5 [1.7, 4.8]	0.1 [-0.4, 2.8]*	3.3 [1.7, 7.0]	2.8 [1.6, 6.6]	0.3 [-0.2, 1.4]**
Brainstem (*D* > 15 Gy) 1% LET	6.0 [2.5, 8.1]	5.0 [2.4, 6.4]	0.1 [-0.2, 3.4]*	6.5 [2.6, 8.9]	5.4 [2.4, 7.9]	0.6 [-0.1, 2.4]**
Pituitary Gland Mean LET	5.0 [2.1, 7.4]	3.2 [2.1, 7.9]	0.8 [-0.5, 2.6]**	5.3 [2.9, 8.2]	4.1 [2.1, 7.5]	0.4 [-0.7, 3.3]**
Pituitary Gland 1% LET	5.8 [2.6, 10.0]	3.6 [2.5, 10.2]	0.8 [-1.5, 3.6]*	7.7 [3.1, 10.7]	5.9 [2.7, 10.3]	0.6 [-1.0, 4.9]*
Ipsilateral Cochlea Mean LET	2.8 [2.2, 3.7]	2.2 [1.8, 3.1]	0.8 [-0.7, 1.5]**	3.1 [2.3, 4.0]	2.4 [1.7, 3.8]	0.6 [-0.3, 1.1]**
Ipsilateral Cochlea 1% LET	3.4 [2.3, 4.8]	2.5 [2.0, 3.4]	1.0 [-0.9, 2.0]**	3.6 [2.8, 5.8]	3 [2.1, 5.3]	0.7 [-0.3, 1.4]***
Brain (*D* > 15 Gy) Mean LET	3 [1.7, 3.7]	2.9 [1.7, 3.5]	0.1 [-0.1, 1.1]*	3.3 [2.5, 3.9]	3.2 [2.5, 4]	0 [-0.1, 0.4]
Brain (*D* > 15 Gy) 1% LET	6.8 [3.5, 8.7]	6.6 [3.5, 8.8]	-0.1 [-1.1, 1.3]	7.3 [4.8, 8.5]	7.2 [5.3, 8]	0 [-0.8, 2.4]

Table A6: Boost plan LET statistics for the GTV and OARs for the median patient and range with differences. Both LET-optimized and non-LET optimized IMPT and PAT plans are shown and the differences between LET and non-LET optimized plans. *p < 0.05, **p < 0.01, ***p < 0.001 from Wilcoxon signed rank test.

LET Statistics [keV/ µm]	IMPT	IMPT-LET	Difference (IMPT – IMPT-LET)	PAT	PAT-LET	Difference (PAT – PAT-LET)
GTV Mean LET	2.8 [2.2, 3.1]	3.5 [2.7, 4.3]	-0.7 [-1.4, -0.2]***	3.0 [2.1, 3.2]	3.6 [2.1, 4.3]	-0.7 [-1.1, 0]**
GTV 1% LET	4 [3, 5.3]	4.7 [4.0, 5.7]	-0.8 [-1.7, 0]**	4.6 [3.4, 5.6]	4.9 [4.0, 5.9]	-0.2 [-1, 0]**
Brainstem (D_s > 5 Gy) Mean LET	4.1 [2.7, 6.8]	2.8 [2.1, 6.1]	0 [-0.1, 2.1]*	4.2 [2.7, 6.1]	3.4 [1.6, 6.3]	0 [-0.2, 2.8]*
Brainstem (*D* > 5 Gy) 1% LET	6.4 [3.3, 7.7]	4.1 [3.3, 6.6]	0 [-0.7, 3.2]	7 [4.2, 8.9]	6 [2.6, 8.7]	0 [-0.1, 2.9]
Pituitary Gland Mean LET	6.3 [1.9, 11.7]	6.3 [1.7, 10.5]	0.3 [-1.2, 5.0]	6.6 [2.5, 10.7]	5.6 [2.4, 11.1]	0.2 [-0.7, 5.1]
Pituitary Gland 1% LET	7.6 [2.1, 13.9]	8.2 [2.3, 16.6]	-0.2 [-5.9, 3.0]	9.7 [3.0, 12.9]	6.6 [3.0, 12.1]	0.8 [-0.2, 5.4]*
Ipsilateral Cochlea Mean LET	3.1 [1.2, 4.5]	2.3 [1.2, 4.9]	0.7 [-0.5, 1.7]*	3.1 [1.4, 7.8]	2.3 [1.5, 5.1]	0.6 [-0.1, 2.8]***
Ipsilateral Cochlea 1% LET	3.7 [1.3, 5.6]	2.9 [1.3, 5.8]	0.8 [-0.5, 2.4]*	3.3 [1.5, 10.8]	2.7 [1.6, 7.8]	0.7 [-0.1, 4.1]**
Brain (*D* > 5 Gy) Mean LET	3.1 [1.3, 4.2]	3 [1.4, 3.9]	0.1 [-0.3, 0.9]	3.5 [2.4, 4.4]	3.6 [2.5, 4.2]	0.1 [-0.6, 0.6]
Brain (*D* > 5 Gy) 1% LET	7.2 [3.5, 8.8]	7.3 [3.6, 9.3]	-0.1 [-2.7, 2.5]	8.5 [5.0, 10.7]	8.3 [4.1, 9.8]	0.3 [-1.3, 2.3]

Table A7: RBE_{LYN}-weighted dose statistics (Variable RBE from the Lyngholm RBE model) for the GTV and OARs for the median patient and range with differences. Both LET-optimized and non-LET optimized IMPT and PAT plans are shown and the differences between LET and non-LET optimized plans. *p < 0.05, **p < 0.01, ***p < 0.001 from Wilcoxon signed rank test. MD: Mean Dose, HI: Homogeneity Index.

Dose Statistics	IMPT	IMPT-LET	Difference	PAT	PAT-LET	Difference
Body MD [Gy(RBE)]	5.98 [2.15, 9.66]	6.19 [2.20, 9.74]	-0.04 [-1.32, -0.01]***	5.61 [2.23, 10.30]	5.60 [2.41, 10.93]	-0.05 [-1.41, 0.17]*
Brain MD [Gy(RBE)]	4.54 [1.26, 7.24]	4.48 [1.26, 8.24]	-0.01 [-2.17, 0.19]	3.93 [1.62, 7.66]	4.59 [1.74, 8.88]	-0.12 [-1.22, 0.37]
Ipsilateral Cochlea MD [Gy(RBE)]	45.04 [12.23, 60.21]	46.86 [13.60, 59.83]	0.71 [-3.77, 3.76]	46.38 [11.97, 62.93]	37.24 [15.22, 60.69]	1.64 [-5.80, 3.45]
Pituitary Gland MD [Gy(RBE)]	40.54 [2.07, 59.99]	39.47 [2.30, 60.68]	-0.12 [-6.19, 6.36]	47.67 [2.10, 66.04]	46.86 [3.20, 63.47]	0.29 [-7.85, 2.81]
Brainstem $D_{0.01cc}$ [Gy(RBE)]	38.10 [3.23, 65.88]	36.36 [3.06, 62.44]	0.45 [-5.94, 10.22]	39.91 [5.75, 64.68]	41.32 [6.84, 62.51]	0.64 [-7.14, 8.81]
Ipsilateral Hippocampus Head MD [Gy(RBE)]	14.45 [1.03, 38.62]	14.10 [0.78, 38.95]	0.24 [-5.38, 1.39]	15.56 [1.48, 36.61]	10.12 [1.49, 37.56]	0.25 [-6.58, 6.99]
Ipsilateral Lens MD [Gy(RBE)]	5.19 [1.15, 19.95]	2.89 [1.28, 21.20]	-0.07 [-1.25, 2.54]	4.02 [0.87, 28.55]	3.86 [1.59, 26.33]	-0.11 [-3.24, 2.22]
GTV D_{50} [Gy(RBE)]	58.87 [56.98, 69.55]	62.85 [61.72, 70.73]	-3.94 [-6.65, -1.76]***	60.16 [58.29, 70.51]	62.94 [61.99, 72.99]	-2.48 [-4.78, 0.57]***
GTV D_{99} [Gy(RBE)]	57.10 [55.72, 65.99]	60.15 [57.14, 66.24]	-2.67 [-5.24, -0.11]***	57.74 [56.93, 67.17]	60.22 [57.23, 67.88]	-2.48 [-4.78, 1.26]**
GTV HI [%]	8.64 [5.88, 17.44]	9.33 [7.71, 16.58]	-0.98 [-6.21, 3.89]	11.97 [8.97, 16.59]	10.57 [6.84, 17.66]	0.93 [-5.85, 3.79]

Table A8: Dose statistics for all five modalities with first standard deviation and colour coded from lowest (blue) to highest (red) dose. MD: mean dose.

	VMAT	IMPT	IMPT-LET	PAT	PAT-LET

Body MD	7.5 ± 3.0	5.1 ± 1.7	5.2 ± 1.8	5.2 ± 2.0	5.3 ± 1.9
Brain MD	5.9 ± 2.7	3.5 ± 1.9	3.7 ± 2.0	3.3 ± 1.5	3.4 ± 1.5
Brainstem D10%	30.9 ± 7.9	15.7 ± 13.2	15.5 ± 13.0	17.8 ± 12.6	18.5 ± 12.6
Brainstem D50%	18.7 ± 10.5	5.7 ± 6.3	5.4 ± 6.1	6.5 ± 6.4	7.1 ± 7.1
Brainstem $D_{0.1cc}$	40.5 ± 10.3	27.4 ± 17.4	27.4 ± 17.3	29.9 ± 16.1	30.3 ± 15.9
Brainstem Core $D_{0.1cc}$	35.9 ± 9.9	19.6 ± 14.7	19.4 ± 14.6	22.3 ± 14.0	23.3 ± 14.2
Ipsilateral Cochlea MD	42.6 ± 12.9	34.4 ± 17.6	35.4 ± 17.9	33.9 ± 18.8	34.8 ± 18.2
Contralateral Cochlea MD	13.6 ± 11.9	9.7 ± 13.2	10.4 ± 14.4	8.7 ± 12.9	8.8 ± 13.2
HippoHead Ipsi MD	12.1 ± 12.8	11.3 ± 8.9	11.8 ± 9.3	11.7 ± 8.9	11.7 ± 8.7
HippoHead Contra MD	3.4 ± 1.8	1.5 ± 1.8	1.7 ± 1.9	0.9 ± 1.7	1.2 ± 2.4
HippoTail Ipsi MD	5.0 ± 6.5	2.4 ± 3.5	2.5 ± 3.5	2.4 ± 3.4	2.4 ± 3.3
HippoTail Contra MD	2.0 ± 0.9	0.2 ± 0.2	0.2 ± 0.3	0.1 ± 0.2	0.2 ± 0.5
Hypothalamus MD	3.4 ± 1.9	2.8 ± 2.9	3.1 ± 4.0	2.1 ± 2.0	2.3 ± 2.1
Lacrimal Ipsi MD	24.0 ± 17.0	18.1 ± 13.7	18.3 ± 13.8	19.1 ± 16.0	19.8 ± 15.7
Lacrimal Contra MD	5.2 ± 4.2	0.8 ± 1.6	0.9 ± 1.9	0.8 ± 1.6	0.7 ± 1.3
Lens Ipsi MD	12.0 ± 9.4	4.6 ± 4.5	4.5 ± 4.7	5.6 ± 6.3	5.6 ± 5.8
Lens Contra MD	3.7 ± 1.5	0.5 ± 0.5	0.5 ± 0.5	0.7 ± 0.8	0.6 ± 0.6
Optic Chiasm $D_{0.1cc}$	15.8 ± 9.8	14.8 ± 12.5	16.3 ± 12.5	14.2 ± 12.5	15.2 ± 12.8
Optic Nerve Ipsi $D_{0.1cc}$	43.5 ± 10.8	40.7 ± 12.0	41.7 ± 11.8	40.2 ± 13.7	40.8 ± 13.4
Optic Nerve Contra $D_{0.1cc}$	29.2 ± 12.4	19.6 ± 15.8	22.4 ± 16.5	15.9 ± 17.3	17.0 ± 17.1
Pituitary MD	37.9 ± 15.3	30.4 ± 17.7	31.7 ± 17.9	29.2 ± 18.5	30.3 ± 18.5
Retina Ipsi $D_{0.1cc}$	36.1 ± 15.1	33.6 ± 12.7	33.7 ± 11.9	33.1 ± 12.8	33.9 ± 12.2
Retina Contra $D_{0.1cc}$	13.0 ± 9.2	6.3 ± 8.1	6.3 ± 8.4	7.0 ± 9.0	6.2 ± 8.4
Spinal Cord $D_{0.1cc}$	22.8 ± 11.9	6.7 ± 6.7	6.3 ± 6.6	6.6 ± 5.6	6.4 ± 4.8
Left Temp Lobe V_{20Gy}	35.5 ± 24.0	34.1 ± 16.2	35.7 ± 15.8	34.9 ± 17.0	35.7 ± 15.8
Right Temp Lobe V_{20Gy}	8.4 ± 6.1	3.2 ± 5.8	3.6 ± 5.8	2.7 ± 5.3	2.6 ± 5.1

Table A9: Mean and 1% LET for the four proton modalities for the initial plans colour coded from lowest (blue) to highest (red) LET. ROIs that had LET criteria applied are shown in bold.

Initial Plan	Mean	1% LET

	IMPT	IMPT-LET	PAT	PAT-LET	IMPT	IMPT-LET	PAT	PAT-LET
Brain (D>15Gy)	3.1 ± 0.5	2.9 ± 0.5	3.3 ± 0.4	3.2 ± 0.5	6.7 ± 1.2	6.5 ± 1.4	7.2 ± 1.1	6.9 ± 0.8
**Brainstem (D>15Gy)**	4.0 ± 1.4	3.3 ± 1.0	3.9 ± 1.5	3.3 ± 1.5	5.9 ± 1.7	4.8 ± 1.4	6.1 ± 1.8	5.3 ± 1.8
**Brainstem Core (D>15Gy)**	4.0 ± 1.4	2.9 ± 0.9	3.5 ± 1.3	2.9 ± 1.1	5.2 ± 1.4	3.9 ± 1.4	5.4 ± 1.7	4.6 ± 1.7
Brain	3.6 ± 0.5	3.5 ± 0.5	3.8 ± 0.5	3.7 ± 0.5	11.2 ± 3.9	10.9 ± 3.9	11.6 ± 3.0	11.7 ± 3.5
**Brainstem**	4.6 ± 1.4	4.0 ± 1.3	4.2 ± 1.3	3.7 ± 1.4	8.1 ± 1.8	8.0 ± 2.3	8.4 ± 2.4	8.0 ± 3.1
**Brainstem Core**	4.5 ± 1.4	3.9 ± 1.3	4.1 ± 1.4	3.6 ± 1.4	7.4 ± 1.8	7.2 ± 2.0	8.0 ± 2.4	7.5 ± 3.1
**Cochlea Ipsi**	2.9 ± 0.5	2.3 ± 0.3	3.1 ± 0.6	2.6 ± 0.6	3.5 ± 0.7	2.6 ± 0.4	3.8 ± 0.9	3.2 ± 0.9
**Optic Nerve Ipsi**	2.9 ± 0.7	2.5 ± 0.5	3.5 ± 0.9	3.0 ± 0.8	4.6 ± 1.6	3.3 ± 0.9	5.9 ± 2.7	4.6 ± 2.2
**Pituitary**	3.9 ± 1.4	3.1 ± 1.0	4.9 ± 1.8	4.1 ± 1.4	4.7 ± 1.9	3.7 ± 1.4	6.6 ± 2.9	5.4 ± 2.3
Retina Ipsi (D>15Gy)	2.7 ± 0.9	2.7 ± 0.8	2.8 ± 0.9	2.6 ± 0.8	4.1 ± 1.7	4.2 ± 1.6	4.1 ± 1.1	3.9 ± 1.3
Retina Ipsi	3.0 ± 1.0	3.0 ± 1.0	2.8 ± 0.6	2.7 ± 0.5	5.1 ± 1.9	5.3 ± 1.8	4.7 ± 1.4	4.8 ± 1.4
CTV1	2.5 ± 0.2	3.2 ± 0.4	2.8 ± 0.2	3.2 ± 0.3	3.8 ± 0.3	4.2 ± 0.4	4.5 ± 0.5	4.6 ± 0.3
**CTV2/GTV**	2.4 ± 0.2	3.3 ± 0.5	2.8 ± 0.2	3.4 ± 0.4	3.2 ± 0.2	4.0 ± 0.5	3.8 ± 0.4	4.3 ± 0.3

Table A10: Mean and 1% LET for the four proton modalities for the boost plans colour coded from lowest (blue) to highest (red) LET. ROIs that had LET criteria applied are shown in bold.

Boost Plan	Mean	1% LET

	IMPT	IMPT-LET	PAT	PAT-LET	IMPT	IMPT-LET	PAT	PAT-LET
Brain (D>5Gy)	3.1 ± 0.7	2.9 ± 0.7	3.5 ± 0.6	3.5 ± 0.6	6.9 ± 1.5	6.8 ± 1.8	8.2 ± 1.4	7.9 ± 1.4
**Brainstem (D>5Gy)**	4.2 ± 1.5	3.2 ± 1.4	4.4 ± 1.2	3.5 ± 1.5	6.0 ± 1.5	4.8 ± 1.3	6.7 ± 2.0	5.5 ± 2.3
**Brainstem Core (D>5Gy)**	4.0 ± 1.1	2.6 ± 0.6	4.4 ± 1.7	3.4 ± 1.9	5.9 ± 0.7	4.4 ± 1.1	6.8 ± 1.8	4.9 ± 2.2
Brain	3.5 ± 0.5	3.3 ± 0.6	3.8 ± 0.6	3.7 ± 0.6	12.6 ± 3.8	12.8 ± 4.0	13.0 ± 3.2	12.7 ± 3.4
**Brainstem**	5.3 ± 1.4	4.4 ± 1.4	5.1 ± 1.5	4.3 ± 1.4	10.7 ± 5.9	11.1 ± 7.5	11.1 ± 5.5	10.2 ± 6.6
**Brainstem Core**	5.3 ± 1.4	4.5 ± 1.3	5.0 ± 1.6	4.2 ± 1.6	10.2 ± 6.2	11.1 ± 8.2	10.9 ± 6.4	9.9 ± 7.2
**Cochlea Ipsi**	3.1 ± 0.8	2.4 ± 0.9	3.7 ± 1.8	2.8 ± 1.1	3.7 ± 1.2	2.8 ± 1.0	4.5 ± 2.6	3.4 ± 1.8
**Optic Nerve Ipsi**	3.8 ± 1.5	3.2 ± 1.5	4.2 ± 1.4	3.4 ± 1.2	7.0 ± 3.6	5.4 ± 3.4	7.8 ± 3.9	6.1 ± 3.5
**Pituitary**	5.1 ± 3.4	4.9 ± 2.8	6.6 ± 3.3	5.6 ± 3.0	6.4 ± 3.9	6.3 ± 3.6	7.8 ± 3.7	6.8 ± 3.4
Retina Ipsi (D>5Gy)	2.9 ± 1.0	3.0 ± 1.3	2.8 ± 0.8	2.6 ± 0.8	3.9 ± 1.0	4.3 ± 1.8	4.1 ± 1.4	3.8 ± 1.5
Retina Ipsi	3.4 ± 1.1	3.2 ± 1.0	3.0 ± 0.7	2.8 ± 0.6	5.1 ± 1.5	5.3 ± 1.8	5.3 ± 1.8	4.8 ± 1.5
CTV1	3.0 ± 0.3	3.5 ± 0.5	3.2 ± 0.4	3.6 ± 0.5	6.3 ± 1.7	6.8 ± 1.3	7.3 ± 1.9	7.0 ± 1.6
**CTV2/GTV**	2.7 ± 0.3	3.5 ± 0.5	2.9 ± 0.3	3.6 ± 0.6	3.9 ± 0.6	4.7 ± 0.7	4.5 ± 0.6	4.8 ± 0.5

Table A11: LET-weighted dose (LWD) statistics for all five modalities with first standard deviation and colour coded from lowest (blue) to highest (red) dose. MD: mean dose.

VMAT	IMPT	IMPT-LET	Arc	Arc-LET

Body	7.5 ± 3.0	5.2 ± 1.8	5.3 ± 1.8	5.3 ± 2.0	5.5 ± 2.1
Brain	5.9 ± 2.7	3.3 ± 1.8	3.7 ± 2.0	3.4 ± 1.6	3.6 ± 1.7
Brainstem D10%	30.9 ± 7.9	16.5 ± 13.7	15.7 ± 12.9	18.5 ± 13.2	19.3 ± 13.3
Brainstem D50%	18.7 ± 10.5	5.9 ± 6.5	5.5 ± 6.1	6.6 ± 6.4	7.2 ± 7.0
Brainstem $D_{0.1cc}$	40.5 ± 10.3	28.9 ± 17.9	28.3 ± 17.6	31.7 ± 17.0	32.1 ± 16.8
brainstemCore	35.9 ± 9.9	20.6 ± 15.2	19.8 ± 14.8	23.6 ± 14.8	24.5 ± 15.0
Cochlea Left	42.6 ± 12.9	34.9 ± 17.8	35.1 ± 17.8	34.6 ± 19.2	34.9 ± 18.2
Cochlea Right	13.6 ± 11.9	10.1 ± 13.5	10.4 ± 14.4	9.1 ± 13.4	9.0 ± 13.2
HippoHead Lt	12.1 ± 12.8	11.4 ± 8.8	11.8 ± 9.1	11.9 ± 9.0	12.4 ± 9.5
HippoHead Rt	3.4 ± 1.8	1.5 ± 1.9	1.9 ± 2.0	1.0 ± 1.7	1.2 ± 2.4
HippoTail Lt	5.0 ± 6.5	2.5 ± 3.6	2.6 ± 3.6	2.5 ± 3.5	2.7 ± 3.9
HippoTail Rt	2.0 ± 0.9	0.2 ± 0.2	0.2 ± 0.3	0.1 ± 0.2	0.2 ± 0.5
Hypothalamus	3.4 ± 1.9	2.9 ± 2.9	3.2 ± 3.8	2.3 ± 2.1	2.5 ± 2.4
Lacrimal Left	24.0 ± 17.0	18.6 ± 14.1	18.9 ± 14.2	19.4 ± 16.4	20.5 ± 16.2
Lacrimal Right	5.2 ± 4.2	0.7 ± 1.6	0.9 ± 1.9	0.7 ± 1.6	0.7 ± 1.3
Lens Left	12.0 ± 9.4	4.6 ± 4.4	4.6 ± 4.7	5.5 ± 6.3	5.6 ± 5.8
Lens Right	3.7 ± 1.5	0.5 ± 0.6	0.5 ± 0.6	0.7 ± 0.8	0.7 ± 0.6
Optic Chiasm	15.8 ± 9.8	15.7 ± 12.7	16.3 ± 12.2	15.7 ± 13.1	16.2 ± 13.0
Optic Nerve Lt	43.5 ± 10.8	41.3 ± 11.9	42.0 ± 11.6	41.4 ± 13.8	42.0 ± 14.1
Optic Nerve Rt	29.2 ± 12.4	20.5 ± 15.9	22.6 ± 16.2	16.6 ± 17.6	18.5 ± 18.3
Pituitary	37.9 ± 15.3	31.9 ± 17.7	32.4 ± 17.7	31.5 ± 18.9	32.2 ± 19.1
Retina Left	36.1 ± 15.1	34.0 ± 13.0	34.2 ± 12.3	33.1 ± 13.1	34.6 ± 12.7
Retina Right	13.0 ± 9.2	6.6 ± 7.9	6.6 ± 8.2	7.5 ± 9.5	6.8 ± 8.9
Spinal Cord	22.8 ± 11.9	7.1 ± 7.0	6.6 ± 6.7	6.7 ± 5.6	6.6 ± 4.7
Left Temp Lobe	35.5 ± 24.0	34.4 ± 16.5	35.8 ± 16.0	35.2 ± 17.3	36.6 ± 17.5
Right Temp Lobe	8.4 ± 6.1	3.3 ± 5.8	3.7 ± 5.7	2.8 ± 5.2	2.8 ± 5.1

Table A12: Variable RBE-weighted dose statistics from the McNamara RBE model (MCN) for all five modalities with first standard deviation and colour coded from lowest (blue) to highest (red) dose. MD: mean dose.

	VMAT	IMPT	IMPT-LET	Arc	Arc-LET
Body	7.5 ± 3.0	5.9 ± 2.0	6.1 ± 2.1	6.1 ± 2.3	6.3 ± 2.4

Brain	5.9 ± 2.7	3.9 ± 2.0	4.3 ± 2.3	4.0 ± 1.9	4.2 ± 2.0
Brainstem D10%	30.9 ± 7.9	19.8 ± 15.5	18.6 ± 14.5	22.0 ± 14.8	22.4 ± 14.7
Brainstem D50%	18.7 ± 10.5	7.5 ± 8.0	6.8 ± 7.1	8.2 ± 7.5	8.7 ± 8.0
Brainstem $D_{0.1cc}$	40.5 ± 10.3	33.3 ± 19.3	32.3 ± 18.8	36.8 ± 17.7	36.9 ± 17.8
brainstemCore	35.9 ± 9.9	24.5 ± 17.0	23.1 ± 16.3	28.0 ± 16.2	28.5 ± 16.4
Cochlea Left	42.6 ± 12.9	38.5 ± 18.8	38.4 ± 18.8	38.4 ± 20.1	38.3 ± 19.1
Cochlea Right	13.6 ± 11.9	11.9 ± 15.6	12.1 ± 16.1	10.9 ± 15.6	10.5 ± 15.0
HippoHead Lt	12.1 ± 12.8	13.6 ± 9.8	13.9 ± 10.2	14.2 ± 10.1	14.7 ± 10.8
HippoHead Rt	3.4 ± 1.8	2.1 ± 2.4	2.5 ± 2.7	1.3 ± 2.1	1.6 ± 2.9
HippoTail Lt	5.0 ± 6.5	3.2 ± 4.3	3.3 ± 4.5	3.2 ± 4.3	3.5 ± 5.0
HippoTail Rt	2.0 ± 0.9	0.2 ± 0.3	0.2 ± 0.4	0.2 ± 0.2	0.3 ± 0.6
Hypothalamus	3.4 ± 1.9	3.8 ± 3.4	3.9 ± 4.4	3.3 ± 2.9	3.5 ± 3.2
Lacrimal Left	24.0 ± 17.0	21.4 ± 15.5	21.8 ± 15.6	21.9 ± 17.9	23.2 ± 17.7
Lacrimal Right	5.2 ± 4.2	0.9 ± 1.8	1.1 ± 2.1	0.9 ± 1.8	0.8 ± 1.5
Lens Left	12.0 ± 9.4	5.9 ± 5.2	5.7 ± 5.5	6.7 ± 7.4	6.7 ± 6.7
Lens Right	3.7 ± 1.5	0.8 ± 0.8	0.8 ± 0.8	1.0 ± 1.1	1.0 ± 0.9
Optic Chiasm	15.8 ± 9.8	19.2 ± 14.4	19.0 ± 13.2	19.8 ± 14.8	19.7 ± 14.4
Optic Nerve Lt	43.5 ± 10.8	45.5 ± 11.6	46.1 ± 11.4	46.0 ± 13.9	46.2 ± 14.2
Optic Nerve Rt	29.2 ± 12.4	24.2 ± 17.2	26.0 ± 17.0	19.6 ± 19.2	21.6 ± 19.9
Pituitary	37.9 ± 15.3	35.8 ± 18.5	36.1 ± 18.5	35.6 ± 20.0	36.0 ± 20.1
Retina Left	36.1 ± 15.1	38.1 ± 13.9	38.3 ± 13.2	37.4 ± 14.1	38.8 ± 13.6
Retina Right	13.0 ± 9.2	8.7 ± 8.9	8.4 ± 9.0	9.4 ± 11.0	8.6 ± 10.1
Spinal Cord	22.8 ± 11.9	9.4 ± 8.4	8.5 ± 7.7	8.7 ± 6.6	8.5 ± 5.3
Left Temp Lobe	35.5 ± 24.0	37.5 ± 17.3	39.0 ± 16.9	38.5 ± 18.1	39.7 ± 18.0
Right Temp Lobe	8.4 ± 6.1	4.1 ± 6.5	4.4 ± 6.3	3.4 ± 5.9	3.4 ± 5.7

Table A13: Variable RBE-weighted dose statistics from the Lyngholm RBE model (LYN) for all five modalities with first standard deviation and colour coded from lowest (blue) to highest (red) dose. MD: mean dose.

	VMAT	IMPT	IMPT-LET	Arc	Arc-LET
Body	7.5 ± 3.0	6.2 ± 2.1	6.4 ± 2.2	6.4 ± 2.4	6.6 ± 2.5
Brain	5.9 ± 2.7	4.3 ± 2.2	4.5 ± 2.4	4.2 ± 2.0	4.5 ± 2.1
Brainstem D10%	30.9 ± 7.9	20.8 ± 16.3	19.6 ± 15.1	23.1 ± 15.5	23.5 ± 15.4

Brainstem D50%	18.7 ± 10.5	7.9 ± 8.4	7.2 ± 7.6	8.7 ± 7.9	9.3 ± 8.5
Brainstem $D_{0.1cc}$	40.5 ± 10.3	34.6 ± 20.3	33.9 ± 19.8	38.8 ± 18.8	38.7 ± 18.8
brainstemCore	35.9 ± 9.9	25.7 ± 17.8	24.3 ± 17.1	29.4 ± 17.0	29.9 ± 17.2
Cochlea Left	42.6 ± 12.9	40.6 ± 19.8	40.3 ± 19.7	40.5 ± 21.2	36.6 ± 18.6
Cochlea Right	13.6 ± 11.9	12.6 ± 16.4	12.7 ± 16.9	11.4 ± 16.4	14.6 ± 22.5
HippoHead Lt	12.1 ± 12.8	14.3 ± 10.2	14.6 ± 10.6	15.0 ± 10.5	14.3 ± 11.9
HippoHead Rt	3.4 ± 1.8	2.2 ± 2.6	2.7 ± 2.8	1.4 ± 2.3	3.0 ± 5.6
HippoTail Lt	5.0 ± 6.5	3.4 ± 4.6	3.5 ± 4.7	3.5 ± 4.6	3.5 ± 5.3
HippoTail Rt	2.0 ± 0.9	0.2 ± 0.3	0.3 ± 0.4	0.2 ± 0.3	0.5 ± 1.2
Hypothalamus	3.4 ± 1.9	4.0 ± 3.6	4.2 ± 4.6	3.5 ± 3.1	3.7 ± 3.4
Lacrimal Left	24.0 ± 17.0	22.6 ± 16.3	23.1 ± 16.4	23.2 ± 18.8	24.3 ± 18.7
Lacrimal Right	5.2 ± 4.2	1.0 ± 2.0	1.2 ± 2.3	1.0 ± 1.9	1.0 ± 1.7
Lens Left	12.0 ± 9.4	6.3 ± 5.5	6.1 ± 5.8	7.2 ± 7.8	7.0 ± 7.3
Lens Right	3.7 ± 1.5	0.8 ± 0.8	0.8 ± 0.8	1.1 ± 1.1	1.3 ± 1.1
Optic Chiasm	15.8 ± 9.8	20.1 ± 15.1	19.9 ± 13.7	20.7 ± 15.6	20.6 ± 15.0
Optic Nerve Lt	43.5 ± 10.8	48.0 ± 12.3	48.5 ± 12.0	48.6 ± 14.7	46.6 ± 16.9
Optic Nerve Rt	29.2 ± 12.4	25.4 ± 18.0	27.1 ± 17.7	20.6 ± 20.2	24.3 ± 21.3
Pituitary	37.9 ± 15.3	37.8 ± 19.5	38.0 ± 19.5	37.7 ± 21.3	38.0 ± 21.2
Retina Left	36.1 ± 15.1	40.0 ± 14.4	40.3 ± 13.8	39.3 ± 14.8	39.0 ± 17.2
Retina Right	13.0 ± 9.2	9.1 ± 9.3	8.9 ± 9.4	9.9 ± 11.6	10.7 ± 11.2
Spinal Cord	22.8 ± 11.9	9.9 ± 8.9	9.0 ± 8.1	9.2 ± 6.9	9.0 ± 5.6
Left Temp Lobe	35.5 ± 24.0	38.5 ± 17.5	40.2 ± 17.2	39.5 ± 18.2	40.8 ± 18.0
Right Temp Lobe	8.4 ± 6.1	4.3 ± 6.8	4.7 ± 6.5	3.6 ± 6.2	3.6 ± 6.1

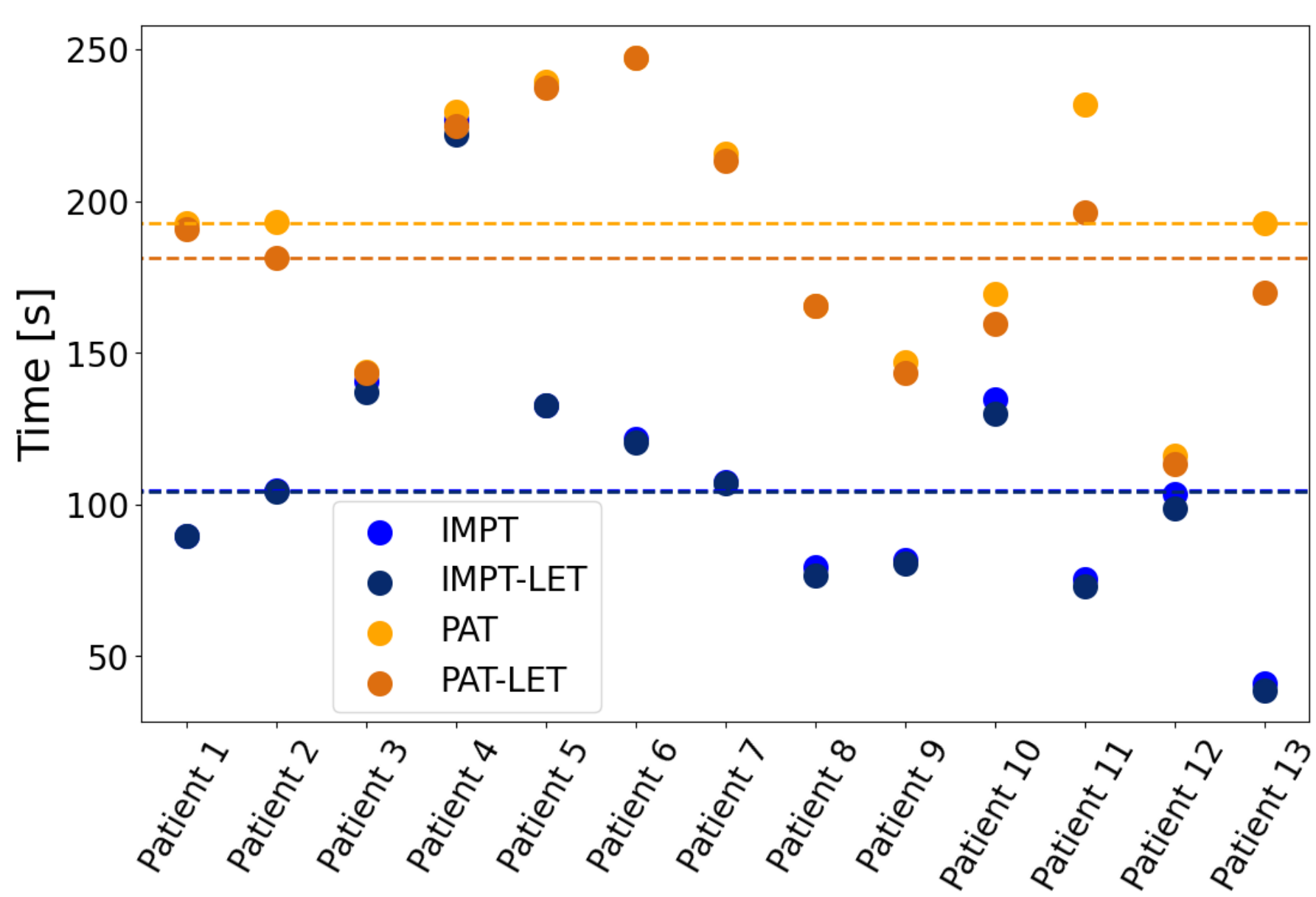

Figure A3: Delivery times for the boost plan for IMPT, LET-optimized IMPT, PAT and LET-optimized PAT for all patients. The median patient is given by the dashed lines.
