# Supplementary material for: Dynamic Proton arc therapy for paediatric parameningeal rhabdomyosarcoma
Source: J Appl Clin Med Phys. 2026 Oct 20;27(11):e70867. doi: 10.1002/acm2.70867 (PMC13682623; doi:10.1002/acm2.70867)
Supplement: Supplementary file 2 [file ACM2-27-e70867-s002.docx]

Supplementary

Figure A1: Tumor locations and adjacent OARs for four example patients.

**Homogeneity Index**

$$HI=\frac{D_{2}-D_{98}}{D_{p}}\cdot100\%$$

(A1)

Here *D*_2_ and *D*_98_ are doses at the specified volume points, approaching maximum and minimum dose, respectively, while *D_p_* is prescribed dose. A low percentage correlates to good homogeneity within the target volume.

**Variable RBE models**

Both the Lyngholm RBE model and the McNamara RBE model are based on the linear-quadratic model formulated as follows (A2):

$$\mathrm{RBE} \left( D_{p}, \left( \frac{\alpha}{\beta} \right)_{x}, \mathrm{RB}E_{\max}, \mathrm{RB}E_{\min} \right)=\frac{1}{2D_{p}}\left( \sqrt{\left( \frac{\alpha}{\beta} \right)_{x}^{2}+4D_{P}\left( \frac{\alpha}{\beta} \right)_{x}\mathrm{RB}E_{\max}+4D_{p}^{2}\mathrm{RB}E_{\min}^{2}}-\left( \frac{\alpha}{\beta} \right)_{x} \right)$$

(A2)

RBE_min_ for the Lyngholm RBE model is given by (A3):

$$\mathrm{RBE}_{\min} =1.021+0.0268\left( \frac{\mathrm{keV}}{\mu m} \right)^{-1}\cdot LET_{d}$$

(A3)

RBE_max_ for the Lyngholm RBE model is given by (A4):

$$\mathrm{RBE}_{\max} =1.161+\frac{0.328\left( \frac{\mathrm{keV}}{\mu m} \right)^{-1}}{\left( \frac{\alpha}{\beta} \right)_{x}}\cdot LET_{d}$$

(A4)

RBE_min_ for the McNamara model is given by (A5):

$$\mathrm{RBE}_{\min} =1.1012-0.0038703\sqrt{\left( \frac{\alpha}{\beta} \right)_{x}}\left( \frac{\mathrm{keV}}{\mu m} \right)^{-1}\cdot LET_{d}$$

(A5)

RBE_max_ for the McNamara RBE model is given by (A6):

$$\mathrm{RBE}_{\max} =0.99064+\frac{0.35605\left( \frac{\mathrm{keV}}{\mu m} \right)^{-1}}{\left( \frac{\alpha}{\beta} \right)_{x}}\cdot LET_{d}$$

(A6)

The LET-weighted dose, or Unkelbach model, describes RBE as a linear function with LET, as follows (A7):

$$\mathrm{RBE} =1+\kappa\cdot LET_{d}$$

(A7)

Where κ is a fitting parameter where a value 0.04 was used as initially proposed by Unkelbach et al.

Table A1: Patient and plan characteristics.

| Patient no. | Prescribed Dose [Gy(RBE)] | Skull Base Laterality |
| --- | --- | --- |
| 1 | 36.0 + 14.4 | Left |
| 2 | 36.0 + 14.4 | Right |
| 3 | 36.0 + 14.4 | Right |
| 4 | 36.0 + 14.4 | Left |
| 5 | 36.0 + 14.4 | Left |
| 6 | 36.0 + 14.4 | Central |
| 7 | 36.0 + 14.4 | Right |
| 8 | 36.0 + 23.4 | Right |
| 9 | 36.0 + 19.8 | Left |
| 10 | 36.0 + 19.8 | Left |
| 11 | 36.0 + 14.4 | Left |
| 12 | 36.0 + 14.4 | Right |
| 13 | 36.0 + 14.4 | Left |

Figure A2: Dose and LET distributions for one example patient including all modalities. a) Dose-distribution VMAT, b) Dose-distribution IMPT, c) LET-distribution IMPT, d) dose-distribution LET-optimized IMPT, e) LET-distribution LET-optimized IMPT, f) dose-distribution PAT, g) LET-distribution PAT, h) dose-distribution LET-optimized PAT, i) LET-distribution LET-optimized PAT.

Table A2: Average RBE_1.1_-weighted dose statistics for the target and OARs between the two dose planners, including the difference between the two planners, for the IMPT plan and the LET-optimized IMPT plan.

| Dose Statistics | IMPT Planner 1 | IMPT Planner 2 | Difference | IMPT-LET Planner 1 | IMPT-LET Planner 2 | Difference |
| --- | --- | --- | --- | --- | --- | --- |
| CTV D50 | 54,3  ±4,4 | 51,2  ±0,3 | 3,0 | 54,7  ±4,4 | 51,9  ±0,5 | 2,8 |
| CTV D99 | 42,8  ±4,7 | 44,2  ±2,7 | -1,5 | 42,6  ±4,6 | 43,7  ±2,0 | -1,1 |
| GTV D50 | 55,4  ±3,9 | 51,6  ±0,2 | 3,8 | 56,3  ±3,5 | 52,4  ±0,3 | 4,0 |
| GTV D99 | 53,4  ±3,0 | 50,7  ±0,1 | 2,7 | 53,6  ±3,0 | 50,7  ±0,1 | 3,0 |
| GTV HI [%] | 7,7  ±1,9 | 3,3  ±0,6 | 0,0 | 8,0  ±1,8 | 5,9  ±1,3 | 2,1 |
| Body | 5,6  ±1,3 | 4,1  ±1,6 | 1,6 | 5,7  ±1,2 | 4,3  ±1,9 | 1,4 |
| Brain | 3,9  ±1,9 | 2,6  ±1,8 | 1,3 | 4,0  ±1,8 | 3,1  ±2,3 | 0,9 |
| Brainstem D10 | 16,0  ±12,1 | 12,0  ±13,4 | 4,0 | 15,4  ±11,3 | 12,3  ±13,8 | 3,2 |
| Brainstem D50 | 5,1  ±5,1 | 4,7  ±6,9 | 0,4 | 4,5  ±4,3 | 4,3  ±6,4 | 0,2 |
| Brainstem D0.01cc | 28,4  ±16,3 | 22,2  ±17,3 | 6,2 | 27,8  ±16,5 | 23,0  ±17,7 | 4,8 |
| brainstemCore | 20,0  ±14,0 | 15,2  ±13,6 | 4,8 | 19,5  ±14,0 | 15,7  ±14,4 | 3,8 |
| Cochlea Left | 38,8  ±13,8 | 32,2  ±15,4 | 6,6 | 39,5  ±13,4 | 33,9  ±16,4 | 5,6 |
| Cochlea Right | 5,0  ±8,7 | 10,0  ±15,8 | -5,1 | 4,9  ±8,9 | 11,4  ±17,7 | -6,5 |
| HippoHead Lt | 11,4  ±11,4 | 11,1  ±7,6 | 0,3 | 11,4  ±11,6 | 12,3  ±8,4 | -0,9 |
| HippoHead Rt | 1,6  ±1,8 | 1,0  ±1,9 | 0,6 | 1,8  ±1,9 | 1,4  ±2,1 | 0,4 |
| HippoTail Lt | 3,5  ±5,0 | 1,5  ±1,4 | 2,0 | 3,5  ±4,9 | 1,7  ±1,9 | 1,8 |
| HippoTail Rt | 0,1  ±0,1 | 0,2  ±0,3 | -0,1 | 0,1  ±0,1 | 0,3  ±0,4 | -0,2 |
| Hypothalamus | 3,2  ±2,0 | 2,3  ±4,0 | 1,0 | 3,4  ±2,3 | 3,0  ±5,7 | 0,5 |
| Lacrimal Left | 15,9  ±11,0 | 15,3  ±11,7 | 0,6 | 16,1  ±11,9 | 15,5  ±11,0 | 0,6 |
| Lacrimal Right | 1,1  ±2,2 | 0,5  ±1,1 | 0,6 | 1,2  ±2,5 | 0,8  ±1,7 | 0,4 |
| Lens Left | 3,5  ±3,5 | 5,7  ±5,7 | -2,2 | 3,5  ±3,6 | 5,6  ±6,2 | -2,0 |
| Lens Right | 0,6  ±0,8 | 0,4  ±0,3 | 0,2 | 0,6  ±0,7 | 0,4  ±0,3 | 0,2 |
| Optic Chiasm | 14,5  ±13,5 | 13,3  ±12,8 | 1,2 | 14,8  ±13,6 | 16,2  ±13,1 | -1,4 |
| Optic Nerve Lt | 42,9  ±9,6 | 36,8  ±14,6 | 6,1 | 42,8  ±10,1 | 38,9  ±14,2 | 3,9 |
| Optic Nerve Rt | 21,0  ±18,9 | 15,6  ±12,9 | 5,4 | 21,6  ±18,5 | 20,4  ±15,9 | 1,2 |
| Pituitary | 33,6  ±17,2 | 23,7  ±17,0 | 9,9 | 33,7  ±17,0 | 26,1  ±18,6 | 7,6 |
| Retina Left | 32,1  ±9,4 | 32,6  ±16,0 | -0,5 | 32,0  ±9,3 | 33,2  ±14,6 | -1,2 |
| Retina Right | 7,3  ±11,5 | 5,4  ±4,6 | 1,9 | 7,4  ±11,9 | 5,0  ±4,9 | 2,4 |
| Spinal Cord | 4,8  ±3,0 | 7,4  ±9,2 | -2,6 | 4,3  ±2,8 | 7,2  ±9,1 | -2,9 |
| Left Temp Lobe | 36,5  ±21,4 | 29,2  ±9,6 | 7,3 | 36,7  ±20,9 | 32,7  ±11,2 | 3,9 |
| Right Temp Lobe | 2,9  ±5,1 | 3,4  ±7,4 | -0,5 | 3,1  ±5,2 | 3,9  ±7,2 | -0,8 |

Table A3: Average RBE_1.1_-weighted dose statistics for the target and OARs between the two dose planners, including the difference between the two planners, for the PAT plan and the LET-optimized PAT plan.

| Dose Statistics | PAT Planner 1 | PAT Planner 2 | Difference | PAT-LET Planner 1 | PAT-LET Planner 2 | Difference |
| --- | --- | --- | --- | --- | --- | --- |
| CTV D50 | 54,7  ±4,4 | 51,6  ±0,9 | 3,1 | 54,7  ±4,4 | 51,6  ±0,9 | 3,1 |
| CTV D99 | 43,0  ±4,8 | 43,1  ±2,8 | -0,1 | 43,0  ±4,7 | 44,2  ±2,5 | -1,3 |
| GTV D50 | 55,9  ±3,7 | 52,1  ±0,7 | 3,8 | 56,6  ±3,7 | 52,3  ±0,3 | 4,4 |
| GTV D99 | 53,7  ±3,1 | 50,9  ±0,4 | 2,8 | 53,7  ±3,1 | 50,7  ±0,2 | 3,0 |
| GTV HI | 8,4  ±0,9 | 4,8  ±1,2 | 3,6 | 8,5  ±1,0 | 5,4  ±0,9 | 3,1 |
| Body | 6,1  ±1,6 | 6,1  ±1,6 | 0,0 | 3,8  ±1,2 | 4,1  ±1,2 | -0,3 |
| Brain | 3,9  ±1,3 | 3,8  ±1,2 | 0,1 | 2,3  ±1,2 | 2,7  ±1,4 | -0,4 |
| Brainstem D10 | 21,6  ±11,2 | 20,8  ±11,9 | 0,8 | 11,0  ±10,9 | 13,5  ±12,4 | -2,5 |
| Brainstem D50 | 7,5  ±5,0 | 6,8  ±5,4 | 0,7 | 3,6  ±5,8 | 5,6  ±8,2 | -2,0 |
| Brainstem D0.01cc | 34,6  ±13,6 | 33,2  ±14,2 | 1,3 | 21,5  ±15,2 | 24,2  ±16,6 | -2,8 |
| brainstemCore | 27,2  ±12,5 | 26,4  ±13,3 | 0,8 | 14,2  ±11,5 | 17,2  ±13,7 | -3,0 |
| Cochlea Left | 38,5  ±16,0 | 38,4  ±16,0 | 0,1 | 31,5  ±16,8 | 33,5  ±15,7 | -1,9 |
| Cochlea Right | 4,9  ±11,0 | 4,8  ±10,9 | 0,0 | 8,7  ±13,7 | 9,3  ±14,6 | -0,6 |
| HippoHead Lt | 13,2  ±10,7 | 13,0  ±10,8 | 0,2 | 9,3  ±7,8 | 9,6  ±7,4 | -0,3 |
| HippoHead Rt | 1,1  ±1,8 | 1,0  ±1,8 | 0,0 | 0,8  ±1,8 | 1,4  ±3,3 | -0,6 |
| HippoTail Lt | 3,8  ±4,7 | 3,5  ±4,6 | 0,3 | 1,2  ±1,0 | 1,4  ±1,2 | -0,2 |
| HippoTail Rt | 0,1  ±0,1 | 0,1  ±0,1 | 0,0 | 0,1  ±0,2 | 0,3  ±0,7 | -0,2 |
| Hypothalamus | 3,2  ±1,9 | 3,4  ±2,0 | -0,2 | 0,7  ±0,7 | 0,8  ±1,0 | -0,1 |
| Lacrimal Left | 19,3  ±15,5 | 19,1  ±15,6 | 0,2 | 14,0  ±12,8 | 15,8  ±13,0 | -1,9 |
| Lacrimal Right | 0,7  ±1,3 | 0,7  ±1,3 | 0,0 | 0,9  ±2,1 | 0,7  ±1,5 | 0,2 |
| Lens Left | 4,1  ±4,4 | 3,8  ±3,8 | 0,3 | 7,0  ±8,4 | 7,3  ±7,7 | -0,3 |
| Lens Right | 0,7  ±0,6 | 0,7  ±0,6 | 0,0 | 0,8  ±1,1 | 0,6  ±0,6 | 0,2 |
| Optic Chiasm | 16,3  ±13,2 | 18,0  ±12,7 | -1,6 | 9,7  ±11,4 | 10,2  ±12,7 | -0,5 |
| Optic Nerve Lt | 45,4  ±6,2 | 46,0  ±6,0 | -0,6 | 33,2  ±17,3 | 33,9  ±16,9 | -0,7 |
| Optic Nerve Rt | 21,7  ±19,3 | 21,4  ±19,5 | 0,4 | 6,4  ±10,0 | 9,3  ±12,1 | -2,9 |
| Pituitary | 34,5  v16,6 | 36,0  ±15,8 | -1,5 | 19,5  ±16,2 | 20,8  ±17,7 | -1,3 |
| Retina Left | 33,8  ±8,5 | 34,0  ±8,3 | -0,2 | 30,0  ±16,4 | 31,5  ±15,8 | -1,6 |
| Retina Right | 9,0  ±12,5 | 8,3  ±11,9 | 0,6 | 4,7  ±5,1 | 3,5  ±3,0 | 1,2 |
| Spinal Cord | 6,7  ±3,7 | 6,5  ±3,3 | 0,2 | 5,7  ±7,6 | 5,5  ±6,2 | 0,2 |
| Left Temp Lobe | 40,2  ±20,5 | 39,6  ±19,0 | 0,5 | 26,9  ±10,6 | 29,2  ±11,1 | -2,3 |
| Right Temp Lobe | 3,2  ±6,2 | 3,2  ±6,2 | 0,0 | 2,3  ±5,3 | 2,1  ±4,8 | 0,2 |

Table A4: Dose statistics for the GTV and OARs for the median patient and range with differences. Both LET-optimized and non-LET optimized IMPT and PAT plans are shown and the differences between LET and non-LET optimized plans. *p < 0.05, **p < 0.01, ***p < 0.001 from Wilcoxon signed rank test. MD: Mean Dose, HI: Homogeneity Index.

| Dose Statistics | IMPT | IMPT-LET | Difference | PAT | PAT-LET | Difference |
| --- | --- | --- | --- | --- | --- | --- |
| Body MD [Gy(RBE)] | 5.03 [1.71, 7.90] | 5.18 [1.75, 7.96] | -0.04 [-1.14,  0]** | 4.71 [1.77,  8.36] | 4.73  [1.90,  8.35] | -0.02 [-0.63,  0.14] |
| Brain MD [Gy(RBE)] | 3.44 [0.90, 6.31] | 3.43 [0.92, 6.93] | -0.02 [-2.05, 0.14] | 3.16 [1.18, 5.91] | 3.79 [1.27, 5.72] | -0.02 [-0.63, 0.28] |
| Ipsilateral Cochlea MD [Gy(RBE)] | 38.27 [9.32, 51.38] | 40.61 [10.91, 51.80] | -0.90 [-5.28, 2.44]* | 36.35 [8.76, 53.84] | 39.85 [12.29,  54.24] | -0.12 [-5.87, 1.52] |
| Pituitary Gland MD [Gy(RBE)] | 29.98 [1.01, 53.28] | 34.29 [1.08, 53.33] | -0.30 [-7.06, 0.72]* | 32.86 [1.21, 55.09] | 36.57 [1.72,  53.63] | -0.28 [-6.86, 1.46] |
| Brainstem *D*_0.01cc_ [Gy(RBE)] | 28.61 [1.56, 52.76] | 28.51 [1.61, 50.96] | 0.03 [-7.72, 8.41] | 29.33 [2.59, 52.01] | 35.10 [3.63, 49.59] | -0.36 [-6.76, 6.97] |
| Ipsilateral Hippocampus Head MD [Gy(RBE)] | 11.30 [0.57, 33.71] | 11.16 [0.46, 34.18] | 0 [-6.16,  0.97] | 11.89 [0.76, 31.01] | 11.52 [0.78,  31.75] | 0.09  [-2.35,  1.78] |
| Ipsilateral Lens MD [Gy(RBE)] | 3.05 [0.86, 15.86] | 1.94 [0.91, 17.14] | -0.03 [-1.28, 1.38] | 2.93 [0.60, 22.83] | 2.82 [0.89, 21.07] | -0.11 [-2.96, 1.89] |
| GTV *D*_50_ [Gy(RBE)] | 52.14 [51.20, 62.01] | 53.48 [52.11,  62.33] | -0.92 [-1.46, -0.22]*** | 52.74 [51.44, 61.94] | 53.66 [52.01, 62.94] | -0.50 [-1.29, 1.00]  * |
| GTV *D*_99_ [Gy(RBE)] | 50.84 [50.58, 57.19] | 50.82 [50.56, 57.00] | 0.03 [-0.72, 0.23] | 51.23 [50.32, 57.64] | 51.03 [50.35, 57.30] | -0.03 [-0.23,  0.66] |
| GTV HI [%] | 5.48 [2.36, 10.23] | 6.45 [4.95,  10.43] | -1.10 [-4.33, 0.79]** | 7.01 [3.50,  9.93] | 7.23 [4.55,  9.91] | -0.35 [-1.78, 1.24] |

Table A5: Initial plan LET statistics for the GTV and OARs for the median patient and range with differences. Both LET-optimized and non-LET optimized IMPT and PAT plans are shown and the differences between LET and non-LET optimized plans. *p < 0.05, **p < 0.01, ***p < 0.001 from Wilcoxon signed rank test.

| LET Statistics [keV/µm] | IMPT | IMPT-LET | Difference (IMPT – IMPT-LET) | PAT | PAT-LET | Difference  (PAT – PAT-LET) |
| --- | --- | --- | --- | --- | --- | --- |
| GTV Mean LET | 2.5 [2.1, 2.8] | 3.4 [2.7, 4.1] | -0.9 [-1.8, -0.2]*** | 2.8 [2.5, 3.1] | 3.4 [2.7, 4.0] | -0.5 [-1.5, 0.1]*** |
| GTV 1% LET | 3.2 [2.8, 3.7] | 4 [3.2, 4.7] | -1 [-1.5, 0.3]** | 3.9 [3.2, 4.6] | 4.2 [3.6, 4.7] | -0.2 [-1.3, 0]** |
| Brainstem (*D* > 15 Gy) Mean LET | 4.1 [1.7, 6.2] | 3.5 [1.7, 4.8] | 0.1 [-0.4, 2.8]* | 3.3 [1.7, 7.0] | 2.8 [1.6, 6.6] | 0.3 [-0.2, 1.4]** |
| Brainstem (*D* > 15 Gy) 1% LET | 6.0 [2.5, 8.1] | 5.0 [2.4, 6.4] | 0.1 [-0.2, 3.4]* | 6.5 [2.6, 8.9] | 5.4 [2.4, 7.9] | 0.6 [-0.1, 2.4]** |
| Pituitary Gland Mean LET | 5.0 [2.1, 7.4] | 3.2 [2.1, 7.9] | 0.8 [-0.5, 2.6]** | 5.3 [2.9, 8.2] | 4.1 [2.1, 7.5] | 0.4 [-0.7, 3.3]** |
| Pituitary Gland 1% LET | 5.8 [2.6, 10.0] | 3.6 [2.5, 10.2] | 0.8 [-1.5, 3.6]* | 7.7 [3.1, 10.7] | 5.9 [2.7,  10.3] | 0.6 [-1.0, 4.9]* |
| Ipsilateral Cochlea Mean LET | 2.8 [2.2, 3.7] | 2.2 [1.8, 3.1] | 0.8 [-0.7, 1.5]** | 3.1 [2.3, 4.0] | 2.4 [1.7, 3.8] | 0.6 [-0.3, 1.1]** |
| Ipsilateral Cochlea 1% LET | 3.4 [2.3, 4.8] | 2.5 [2.0, 3.4] | 1.0 [-0.9, 2.0]** | 3.6 [2.8, 5.8] | 3 [2.1, 5.3] | 0.7 [-0.3, 1.4]*** |
| Brain (*D* > 15 Gy) Mean LET | 3 [1.7, 3.7] | 2.9 [1.7, 3.5] | 0.1 [-0.1, 1.1]* | 3.3 [2.5, 3.9] | 3.2 [2.5, 4] | 0 [-0.1, 0.4] |
| Brain (*D* > 15 Gy) 1% LET | 6.8 [3.5, 8.7] | 6.6 [3.5, 8.8] | -0.1 [-1.1, 1.3] | 7.3 [4.8, 8.5] | 7.2 [5.3,  8] | 0 [-0.8, 2.4] |

Table A6: Boost plan LET statistics for the GTV and OARs for the median patient and range with differences. Both LET-optimized and non-LET optimized IMPT and PAT plans are shown and the differences between LET and non-LET optimized plans. *p < 0.05, **p < 0.01, ***p < 0.001 from Wilcoxon signed rank test.

| LET Statistics [keV/ µm] | IMPT | IMPT-LET | Difference (IMPT – IMPT-LET) | PAT | PAT-LET | Difference (PAT – PAT-LET) |
| --- | --- | --- | --- | --- | --- | --- |
| GTV Mean LET | 2.8 [2.2, 3.1] | 3.5 [2.7, 4.3] | -0.7 [-1.4, -0.2]*** | 3.0 [2.1, 3.2] | 3.6 [2.1, 4.3] | -0.7 [-1.1, 0]** |
| GTV 1% LET | 4 [3, 5.3] | 4.7 [4.0, 5.7] | -0.8 [-1.7, 0]** | 4.6 [3.4, 5.6] | 4.9 [4.0, 5.9] | -0.2 [-1, 0]** |
| Brainstem (*Ds* > 5 Gy) Mean LET | 4.1 [2.7, 6.8] | 2.8 [2.1, 6.1] | 0 [-0.1, 2.1]* | 4.2 [2.7, 6.1] | 3.4 [1.6, 6.3] | 0 [-0.2, 2.8]* |
| Brainstem (*D* > 5 Gy) 1% LET | 6.4 [3.3, 7.7] | 4.1 [3.3, 6.6] | 0 [-0.7, 3.2] | 7 [4.2, 8.9] | 6 [2.6, 8.7] | 0 [-0.1, 2.9] |
| Pituitary Gland Mean LET | 6.3 [1.9, 11.7] | 6.3 [1.7, 10.5] | 0.3 [-1.2, 5.0] | 6.6 [2.5, 10.7] | 5.6 [2.4, 11.1] | 0.2 [-0.7, 5.1] |
| Pituitary Gland 1% LET | 7.6 [2.1, 13.9] | 8.2 [2.3, 16.6] | -0.2 [-5.9, 3.0] | 9.7 [3.0, 12.9] | 6.6 [3.0, 12.1] | 0.8 [-0.2, 5.4]* |
| Ipsilateral Cochlea Mean LET | 3.1 [1.2, 4.5] | 2.3 [1.2, 4.9] | 0.7 [-0.5, 1.7]* | 3.1 [1.4, 7.8] | 2.3 [1.5, 5.1] | 0.6 [-0.1, 2.8]*** |
| Ipsilateral Cochlea 1% LET | 3.7 [1.3, 5.6] | 2.9 [1.3, 5.8] | 0.8 [-0.5, 2.4]* | 3.3 [1.5, 10.8] | 2.7 [1.6, 7.8] | 0.7 [-0.1, 4.1]** |
| Brain (*D* > 5 Gy) Mean LET | 3.1 [1.3, 4.2] | 3 [1.4, 3.9] | 0.1 [-0.3, 0.9] | 3.5 [2.4, 4.4] | 3.6 [2.5, 4.2] | 0.1 [-0.6, 0.6] |
| Brain (*D* > 5 Gy) 1% LET | 7.2 [3.5, 8.8] | 7.3 [3.6, 9.3] | -0.1 [-2.7, 2.5] | 8.5 [5.0, 10.7] | 8.3 [4.1, 9.8] | 0.3 [-1.3, 2.3] |

Table A7: RBELYN-weighted dose statistics (Variable RBE from the Lyngholm RBE model) for the GTV and OARs for the median patient and range with differences. Both LET-optimized and non-LET optimized IMPT and PAT plans are shown and the differences between LET and non-LET optimized plans. *p < 0.05, **p < 0.01, ***p < 0.001 from Wilcoxon signed rank test. MD: Mean Dose, HI: Homogeneity Index.

| Dose Statistics | IMPT | IMPT-LET | Difference | PAT | PAT-LET | Difference |
| --- | --- | --- | --- | --- | --- | --- |
| Body MD [Gy(RBE)] | 5.98 [2.15, 9.66] | 6.19 [2.20, 9.74] | -0.04 [-1.32,  -0.01]*** | 5.61 [2.23,  10.30] | 5.60  [2.41,  10.93] | -0.05 [-1.41,  0.17]* |
| Brain MD [Gy(RBE)] | 4.54 [1.26, 7.24] | 4.48 [1.26, 8.24] | -0.01 [-2.17, 0.19] | 3.93 [1.62, 7.66] | 4.59 [1.74, 8.88] | -0.12 [-1.22, 0.37] |
| Ipsilateral Cochlea MD [Gy(RBE)] | 45.04 [12.23, 60.21] | 46.86 [13.60, 59.83] | 0.71 [-3.77, 3.76] | 46.38 [11.97, 62.93] | 37.24 [15.22,  60.69] | 1.64 [-5.80, 3.45] |
| Pituitary Gland MD [Gy(RBE)] | 40.54 [2.07, 59.99] | 39.47 [2.30, 60.68] | -0.12 [-6.19, 6.36] | 47.67 [2.10, 66.04] | 46.86 [3.20,  63.47] | 0.29 [-7.85, 2.81] |
| Brainstem *D*0.01cc [Gy(RBE)] | 38.10 [3.23, 65.88] | 36.36 [3.06, 62.44] | 0.45 [-5.94, 10.22] | 39.91 [5.75, 64.68] | 41.32 [6.84, 62.51] | 0.64 [-7.14, 8.81] |
| Ipsilateral Hippocampus Head MD [Gy(RBE)] | 14.45 [1.03, 38.62] | 14.10 [0.78, 38.95] | 0.24 [-5.38,  1.39] | 15.56 [1.48, 36.61] | 10.12 [1.49,  37.56] | 0.25  [-6.58,  6.99] |
| Ipsilateral Lens MD [Gy(RBE)] | 5.19 [1.15, 19.95] | 2.89 [1.28, 21.20] | -0.07 [-1.25, 2.54] | 4.02 [0.87, 28.55] | 3.86 [1.59, 26.33] | -0.11 [-3.24, 2.22] |
| GTV *D*50 [Gy(RBE)] | 58.87 [56.98, 69.55] | 62.85 [61.72,  70.73] | -3.94 [-6.65, -1.76]*** | 60.16 [58.29, 70.51] | 62.94 [61.99, 72.99] | -2.48 [-4.78, 0.57]*** |
| GTV *D*99 [Gy(RBE)] | 57.10 [55.72, 65.99] | 60.15 [57.14, 66.24] | -2.67 [-5.24, -0.11]*** | 57.74 [56.93, 67.17] | 60.22 [57.23, 67.88] | -2.48 [-4.78,  1.26]** |
| GTV HI [%] | 8.64 [5.88, 17.44] | 9.33 [7.71,  16.58] | -0.98 [-6.21, 3.89] | 11.97 [8.97,  16.59] | 10.57 [6.84,  17.66] | 0.93 [-5.85, 3.79] |

Table A8: Dose statistics for all five modalities with first standard deviation and colour coded from lowest (blue) to highest (red) dose. MD: mean dose.

|  | VMAT | IMPT | IMPT-LET | PAT | PAT-LET |
| --- | --- | --- | --- | --- | --- |
| Body MD | 7.5 ± 3.0 | 5.1 ± 1.7 | 5.2 ± 1.8 | 5.2 ± 2.0 | 5.3 ± 1.9 |
| Brain MD | 5.9 ± 2.7 | 3.5 ± 1.9 | 3.7 ± 2.0 | 3.3 ± 1.5 | 3.4 ± 1.5 |
| Brainstem D10% | 30.9 ± 7.9 | 15.7 ± 13.2 | 15.5 ± 13.0 | 17.8 ± 12.6 | 18.5 ± 12.6 |
| Brainstem D50% | 18.7 ± 10.5 | 5.7 ± 6.3 | 5.4 ± 6.1 | 6.5 ± 6.4 | 7.1 ± 7.1 |
| Brainstem D_0.1cc_ | 40.5 ± 10.3 | 27.4 ± 17.4 | 27.4 ± 17.3 | 29.9 ± 16.1 | 30.3 ± 15.9 |
| Brainstem Core D_0.1cc_ | 35.9 ± 9.9 | 19.6 ± 14.7 | 19.4 ± 14.6 | 22.3 ± 14.0 | 23.3 ± 14.2 |
| Ipsilateral Cochlea MD | 42.6 ± 12.9 | 34.4 ± 17.6 | 35.4 ± 17.9 | 33.9 ± 18.8 | 34.8 ± 18.2 |
| Contralateral Cochlea MD | 13.6 ± 11.9 | 9.7 ± 13.2 | 10.4 ± 14.4 | 8.7 ± 12.9 | 8.8 ± 13.2 |
| HippoHead Ipsi MD | 12.1 ± 12.8 | 11.3 ± 8.9 | 11.8 ± 9.3 | 11.7 ± 8.9 | 11.7 ± 8.7 |
| HippoHead Contra MD | 3.4 ± 1.8 | 1.5 ± 1.8 | 1.7 ± 1.9 | 0.9 ± 1.7 | 1.2 ± 2.4 |
| HippoTail Ipsi MD | 5.0 ± 6.5 | 2.4 ± 3.5 | 2.5 ± 3.5 | 2.4 ± 3.4 | 2.4 ± 3.3 |
| HippoTail Contra MD | 2.0 ± 0.9 | 0.2 ± 0.2 | 0.2 ± 0.3 | 0.1 ± 0.2 | 0.2 ± 0.5 |
| Hypothalamus MD | 3.4 ± 1.9 | 2.8 ± 2.9 | 3.1 ± 4.0 | 2.1 ± 2.0 | 2.3 ± 2.1 |
| Lacrimal Ipsi MD | 24.0 ± 17.0 | 18.1 ± 13.7 | 18.3 ± 13.8 | 19.1 ± 16.0 | 19.8 ± 15.7 |
| Lacrimal Contra MD | 5.2 ± 4.2 | 0.8 ± 1.6 | 0.9 ± 1.9 | 0.8 ± 1.6 | 0.7 ± 1.3 |
| Lens Ipsi MD | 12.0 ± 9.4 | 4.6 ± 4.5 | 4.5 ± 4.7 | 5.6 ± 6.3 | 5.6 ± 5.8 |
| Lens Contra MD | 3.7 ± 1.5 | 0.5 ± 0.5 | 0.5 ± 0.5 | 0.7 ± 0.8 | 0.6 ± 0.6 |
| Optic Chiasm D_0.1cc_ | 15.8 ± 9.8 | 14.8 ± 12.5 | 16.3 ± 12.5 | 14.2 ± 12.5 | 15.2 ± 12.8 |
| Optic Nerve Ipsi D_0.1cc_ | 43.5 ± 10.8 | 40.7 ± 12.0 | 41.7 ± 11.8 | 40.2 ± 13.7 | 40.8 ± 13.4 |
| Optic Nerve Contra D_0.1cc_ | 29.2 ± 12.4 | 19.6 ± 15.8 | 22.4 ± 16.5 | 15.9 ± 17.3 | 17.0 ± 17.1 |
| Pituitary MD | 37.9 ± 15.3 | 30.4 ± 17.7 | 31.7 ± 17.9 | 29.2 ± 18.5 | 30.3 ± 18.5 |
| Retina Ipsi D_0.1cc_ | 36.1 ± 15.1 | 33.6 ± 12.7 | 33.7 ± 11.9 | 33.1 ± 12.8 | 33.9 ± 12.2 |
| Retina Contra D_0.1cc_ | 13.0 ± 9.2 | 6.3 ± 8.1 | 6.3 ± 8.4 | 7.0 ± 9.0 | 6.2 ± 8.4 |
| Spinal Cord D_0.1cc_ | 22.8 ± 11.9 | 6.7 ± 6.7 | 6.3 ± 6.6 | 6.6 ± 5.6 | 6.4 ± 4.8 |
| Left Temp Lobe V_20Gy_ | 35.5 ± 24.0 | 34.1 ± 16.2 | 35.7 ± 15.8 | 34.9 ± 17.0 | 35.7 ± 15.8 |
| Right Temp Lobe V_20Gy_ | 8.4 ± 6.1 | 3.2 ± 5.8 | 3.6 ± 5.8 | 2.7 ± 5.3 | 2.6 ± 5.1 |

Table A9: Mean and 1% LET for the four proton modalities for the initial plans colour coded from lowest (blue) to highest (red) LET. ROIs that had LET criteria applied are shown in bold.

| Initial Plan | Mean | | | | 1% LET | | | |
| --- | --- | --- | --- | --- | --- | --- | --- | --- |
|  | IMPT | IMPT-LET | PAT | PAT-LET | IMPT | IMPT-LET | PAT | PAT-LET |
| Brain (D>15Gy) | 3.1 ± 0.5 | 2.9 ± 0.5 | 3.3 ± 0.4 | 3.2 ± 0.5 | 6.7 ± 1.2 | 6.5 ± 1.4 | 7.2 ± 1.1 | 6.9 ± 0.8 |
| **Brainstem (D>15Gy)** | 4.0 ± 1.4 | 3.3 ± 1.0 | 3.9 ± 1.5 | 3.3 ± 1.5 | 5.9 ± 1.7 | 4.8 ± 1.4 | 6.1 ± 1.8 | 5.3 ± 1.8 |
| **Brainstem Core (D>15Gy)** | 4.0 ± 1.4 | 2.9 ± 0.9 | 3.5 ± 1.3 | 2.9 ± 1.1 | 5.2 ± 1.4 | 3.9 ± 1.4 | 5.4 ± 1.7 | 4.6 ± 1.7 |
| Brain | 3.6 ± 0.5 | 3.5 ± 0.5 | 3.8 ± 0.5 | 3.7 ± 0.5 | 11.2 ± 3.9 | 10.9 ± 3.9 | 11.6 ± 3.0 | 11.7 ± 3.5 |
| **Brainstem** | 4.6 ± 1.4 | 4.0 ± 1.3 | 4.2 ± 1.3 | 3.7 ± 1.4 | 8.1 ± 1.8 | 8.0 ± 2.3 | 8.4 ± 2.4 | 8.0 ± 3.1 |
| **Brainstem Core** | 4.5 ± 1.4 | 3.9 ± 1.3 | 4.1 ± 1.4 | 3.6 ± 1.4 | 7.4 ± 1.8 | 7.2 ± 2.0 | 8.0 ± 2.4 | 7.5 ± 3.1 |
| **Cochlea Ipsi** | 2.9 ± 0.5 | 2.3 ± 0.3 | 3.1 ± 0.6 | 2.6 ± 0.6 | 3.5 ± 0.7 | 2.6 ± 0.4 | 3.8 ± 0.9 | 3.2 ± 0.9 |
| **Optic Nerve Ipsi** | 2.9 ± 0.7 | 2.5 ± 0.5 | 3.5 ± 0.9 | 3.0 ± 0.8 | 4.6 ± 1.6 | 3.3 ± 0.9 | 5.9 ± 2.7 | 4.6 ± 2.2 |
| **Pituitary** | 3.9 ± 1.4 | 3.1 ± 1.0 | 4.9 ± 1.8 | 4.1 ± 1.4 | 4.7 ± 1.9 | 3.7 ± 1.4 | 6.6 ± 2.9 | 5.4 ± 2.3 |
| Retina Ipsi (D>15Gy) | 2.7 ± 0.9 | 2.7 ± 0.8 | 2.8 ± 0.9 | 2.6 ± 0.8 | 4.1 ± 1.7 | 4.2 ± 1.6 | 4.1 ± 1.1 | 3.9 ± 1.3 |
| Retina Ipsi | 3.0 ± 1.0 | 3.0 ± 1.0 | 2.8 ± 0.6 | 2.7 ± 0.5 | 5.1 ± 1.9 | 5.3 ± 1.8 | 4.7 ± 1.4 | 4.8 ± 1.4 |
| CTV1 | 2.5 ± 0.2 | 3.2 ± 0.4 | 2.8 ± 0.2 | 3.2 ± 0.3 | 3.8 ± 0.3 | 4.2 ± 0.4 | 4.5 ± 0.5 | 4.6 ± 0.3 |
| **CTV2/GTV** | 2.4 ± 0.2 | 3.3 ± 0.5 | 2.8 ± 0.2 | 3.4 ± 0.4 | 3.2 ± 0.2 | 4.0 ± 0.5 | 3.8 ± 0.4 | 4.3 ± 0.3 |

Table A10: Mean and 1% LET for the four proton modalities for the boost plans colour coded from lowest (blue) to highest (red) LET. ROIs that had LET criteria applied are shown in bold.

| Boost Plan | Mean | | | | 1% LET | | | |
| --- | --- | --- | --- | --- | --- | --- | --- | --- |
|  | IMPT | IMPT-LET | PAT | PAT-LET | IMPT | IMPT-LET | PAT | PAT-LET |
| Brain (D>5Gy) | 3.1 ± 0.7 | 2.9 ± 0.7 | 3.5 ± 0.6 | 3.5 ± 0.6 | 6.9 ± 1.5 | 6.8 ± 1.8 | 8.2 ± 1.4 | 7.9 ± 1.4 |
| **Brainstem (D>5Gy)** | 4.2 ± 1.5 | 3.2 ± 1.4 | 4.4 ± 1.2 | 3.5 ± 1.5 | 6.0 ± 1.5 | 4.8 ± 1.3 | 6.7 ± 2.0 | 5.5 ± 2.3 |
| **Brainstem Core (D>5Gy)** | 4.0 ± 1.1 | 2.6 ± 0.6 | 4.4 ± 1.7 | 3.4 ± 1.9 | 5.9 ± 0.7 | 4.4 ± 1.1 | 6.8 ± 1.8 | 4.9 ± 2.2 |
| Brain | 3.5 ± 0.5 | 3.3 ± 0.6 | 3.8 ± 0.6 | 3.7 ± 0.6 | 12.6 ± 3.8 | 12.8 ± 4.0 | 13.0 ± 3.2 | 12.7 ± 3.4 |
| **Brainstem** | 5.3 ± 1.4 | 4.4 ± 1.4 | 5.1 ± 1.5 | 4.3 ± 1.4 | 10.7 ± 5.9 | 11.1 ± 7.5 | 11.1 ± 5.5 | 10.2 ± 6.6 |
| **Brainstem Core** | 5.3 ± 1.4 | 4.5 ± 1.3 | 5.0 ± 1.6 | 4.2 ± 1.6 | 10.2 ± 6.2 | 11.1 ± 8.2 | 10.9 ± 6.4 | 9.9 ± 7.2 |
| **Cochlea Ipsi** | 3.1 ± 0.8 | 2.4 ± 0.9 | 3.7 ± 1.8 | 2.8 ± 1.1 | 3.7 ± 1.2 | 2.8 ± 1.0 | 4.5 ± 2.6 | 3.4 ± 1.8 |
| **Optic Nerve Ipsi** | 3.8 ± 1.5 | 3.2 ± 1.5 | 4.2 ± 1.4 | 3.4 ± 1.2 | 7.0 ± 3.6 | 5.4 ± 3.4 | 7.8 ± 3.9 | 6.1 ± 3.5 |
| **Pituitary** | 5.1 ± 3.4 | 4.9 ± 2.8 | 6.6 ± 3.3 | 5.6 ± 3.0 | 6.4 ± 3.9 | 6.3 ± 3.6 | 7.8 ± 3.7 | 6.8 ± 3.4 |
| Retina Ipsi (D>5Gy) | 2.9 ± 1.0 | 3.0 ± 1.3 | 2.8 ± 0.8 | 2.6 ± 0.8 | 3.9 ± 1.0 | 4.3 ± 1.8 | 4.1 ± 1.4 | 3.8 ± 1.5 |
| Retina Ipsi | 3.4 ± 1.1 | 3.2 ± 1.0 | 3.0 ± 0.7 | 2.8 ± 0.6 | 5.1 ± 1.5 | 5.3 ± 1.8 | 5.3 ± 1.8 | 4.8 ± 1.5 |
| CTV1 | 3.0 ± 0.3 | 3.5 ± 0.5 | 3.2 ± 0.4 | 3.6 ± 0.5 | 6.3 ± 1.7 | 6.8 ± 1.3 | 7.3 ± 1.9 | 7.0 ± 1.6 |
| **CTV2/GTV** | 2.7 ± 0.3 | 3.5 ± 0.5 | 2.9 ± 0.3 | 3.6 ± 0.6 | 3.9 ± 0.6 | 4.7 ± 0.7 | 4.5 ± 0.6 | 4.8 ± 0.5 |

Table A11: LET-weighted dose (LWD) statistics for all five modalities with first standard deviation and colour coded from lowest (blue) to highest (red) dose. MD: mean dose.

|  | VMAT | IMPT | IMPT-LET | Arc | Arc-LET |
| --- | --- | --- | --- | --- | --- |
| Body | 7.5 ± 3.0 | 5.2 ± 1.8 | 5.3 ± 1.8 | 5.3 ± 2.0 | 5.5 ± 2.1 |
| Brain | 5.9 ± 2.7 | 3.3 ± 1.8 | 3.7 ± 2.0 | 3.4 ± 1.6 | 3.6 ± 1.7 |
| Brainstem D10% | 30.9 ± 7.9 | 16.5 ± 13.7 | 15.7 ± 12.9 | 18.5 ± 13.2 | 19.3 ± 13.3 |
| Brainstem D50% | 18.7 ± 10.5 | 5.9 ± 6.5 | 5.5 ± 6.1 | 6.6 ± 6.4 | 7.2 ± 7.0 |
| Brainstem D_0.1cc_ | 40.5 ± 10.3 | 28.9 ± 17.9 | 28.3 ± 17.6 | 31.7 ± 17.0 | 32.1 ± 16.8 |
| brainstemCore | 35.9 ± 9.9 | 20.6 ± 15.2 | 19.8 ± 14.8 | 23.6 ± 14.8 | 24.5 ± 15.0 |
| Cochlea Left | 42.6 ± 12.9 | 34.9 ± 17.8 | 35.1 ± 17.8 | 34.6 ± 19.2 | 34.9 ± 18.2 |
| Cochlea Right | 13.6 ± 11.9 | 10.1 ± 13.5 | 10.4 ± 14.4 | 9.1 ± 13.4 | 9.0 ± 13.2 |
| HippoHead Lt | 12.1 ± 12.8 | 11.4 ± 8.8 | 11.8 ± 9.1 | 11.9 ± 9.0 | 12.4 ± 9.5 |
| HippoHead Rt | 3.4 ± 1.8 | 1.5 ± 1.9 | 1.9 ± 2.0 | 1.0 ± 1.7 | 1.2 ± 2.4 |
| HippoTail Lt | 5.0 ± 6.5 | 2.5 ± 3.6 | 2.6 ± 3.6 | 2.5 ± 3.5 | 2.7 ± 3.9 |
| HippoTail Rt | 2.0 ± 0.9 | 0.2 ± 0.2 | 0.2 ± 0.3 | 0.1 ± 0.2 | 0.2 ± 0.5 |
| Hypothalamus | 3.4 ± 1.9 | 2.9 ± 2.9 | 3.2 ± 3.8 | 2.3 ± 2.1 | 2.5 ± 2.4 |
| Lacrimal Left | 24.0 ± 17.0 | 18.6 ± 14.1 | 18.9 ± 14.2 | 19.4 ± 16.4 | 20.5 ± 16.2 |
| Lacrimal Right | 5.2 ± 4.2 | 0.7 ± 1.6 | 0.9 ± 1.9 | 0.7 ± 1.6 | 0.7 ± 1.3 |
| Lens Left | 12.0 ± 9.4 | 4.6 ± 4.4 | 4.6 ± 4.7 | 5.5 ± 6.3 | 5.6 ± 5.8 |
| Lens Right | 3.7 ± 1.5 | 0.5 ± 0.6 | 0.5 ± 0.6 | 0.7 ± 0.8 | 0.7 ± 0.6 |
| Optic Chiasm | 15.8 ± 9.8 | 15.7 ± 12.7 | 16.3 ± 12.2 | 15.7 ± 13.1 | 16.2 ± 13.0 |
| Optic Nerve Lt | 43.5 ± 10.8 | 41.3 ± 11.9 | 42.0 ± 11.6 | 41.4 ± 13.8 | 42.0 ± 14.1 |
| Optic Nerve Rt | 29.2 ± 12.4 | 20.5 ± 15.9 | 22.6 ± 16.2 | 16.6 ± 17.6 | 18.5 ± 18.3 |
| Pituitary | 37.9 ± 15.3 | 31.9 ± 17.7 | 32.4 ± 17.7 | 31.5 ± 18.9 | 32.2 ± 19.1 |
| Retina Left | 36.1 ± 15.1 | 34.0 ± 13.0 | 34.2 ± 12.3 | 33.1 ± 13.1 | 34.6 ± 12.7 |
| Retina Right | 13.0 ± 9.2 | 6.6 ± 7.9 | 6.6 ± 8.2 | 7.5 ± 9.5 | 6.8 ± 8.9 |
| Spinal Cord | 22.8 ± 11.9 | 7.1 ± 7.0 | 6.6 ± 6.7 | 6.7 ± 5.6 | 6.6 ± 4.7 |
| Left Temp Lobe | 35.5 ± 24.0 | 34.4 ± 16.5 | 35.8 ± 16.0 | 35.2 ± 17.3 | 36.6 ± 17.5 |
| Right Temp Lobe | 8.4 ± 6.1 | 3.3 ± 5.8 | 3.7 ± 5.7 | 2.8 ± 5.2 | 2.8 ± 5.1 |

Table A12: Variable RBE-weighted dose statistics from the McNamara RBE model (MCN) for all five modalities with first standard deviation and colour coded from lowest (blue) to highest (red) dose. MD: mean dose.

|  | VMAT | IMPT | IMPT-LET | Arc | Arc-LET |
| --- | --- | --- | --- | --- | --- |
| Body | 7.5 ± 3.0 | 5.9 ± 2.0 | 6.1 ± 2.1 | 6.1 ± 2.3 | 6.3 ± 2.4 |
| Brain | 5.9 ± 2.7 | 3.9 ± 2.0 | 4.3 ± 2.3 | 4.0 ± 1.9 | 4.2 ± 2.0 |
| Brainstem D10% | 30.9 ± 7.9 | 19.8 ± 15.5 | 18.6 ± 14.5 | 22.0 ± 14.8 | 22.4 ± 14.7 |
| Brainstem D50% | 18.7 ± 10.5 | 7.5 ± 8.0 | 6.8 ± 7.1 | 8.2 ± 7.5 | 8.7 ± 8.0 |
| Brainstem D_0.1cc_ | 40.5 ± 10.3 | 33.3 ± 19.3 | 32.3 ± 18.8 | 36.8 ± 17.7 | 36.9 ± 17.8 |
| brainstemCore | 35.9 ± 9.9 | 24.5 ± 17.0 | 23.1 ± 16.3 | 28.0 ± 16.2 | 28.5 ± 16.4 |
| Cochlea Left | 42.6 ± 12.9 | 38.5 ± 18.8 | 38.4 ± 18.8 | 38.4 ± 20.1 | 38.3 ± 19.1 |
| Cochlea Right | 13.6 ± 11.9 | 11.9 ± 15.6 | 12.1 ± 16.1 | 10.9 ± 15.6 | 10.5 ± 15.0 |
| HippoHead Lt | 12.1 ± 12.8 | 13.6 ± 9.8 | 13.9 ± 10.2 | 14.2 ± 10.1 | 14.7 ± 10.8 |
| HippoHead Rt | 3.4 ± 1.8 | 2.1 ± 2.4 | 2.5 ± 2.7 | 1.3 ± 2.1 | 1.6 ± 2.9 |
| HippoTail Lt | 5.0 ± 6.5 | 3.2 ± 4.3 | 3.3 ± 4.5 | 3.2 ± 4.3 | 3.5 ± 5.0 |
| HippoTail Rt | 2.0 ± 0.9 | 0.2 ± 0.3 | 0.2 ± 0.4 | 0.2 ± 0.2 | 0.3 ± 0.6 |
| Hypothalamus | 3.4 ± 1.9 | 3.8 ± 3.4 | 3.9 ± 4.4 | 3.3 ± 2.9 | 3.5 ± 3.2 |
| Lacrimal Left | 24.0 ± 17.0 | 21.4 ± 15.5 | 21.8 ± 15.6 | 21.9 ± 17.9 | 23.2 ± 17.7 |
| Lacrimal Right | 5.2 ± 4.2 | 0.9 ± 1.8 | 1.1 ± 2.1 | 0.9 ± 1.8 | 0.8 ± 1.5 |
| Lens Left | 12.0 ± 9.4 | 5.9 ± 5.2 | 5.7 ± 5.5 | 6.7 ± 7.4 | 6.7 ± 6.7 |
| Lens Right | 3.7 ± 1.5 | 0.8 ± 0.8 | 0.8 ± 0.8 | 1.0 ± 1.1 | 1.0 ± 0.9 |
| Optic Chiasm | 15.8 ± 9.8 | 19.2 ± 14.4 | 19.0 ± 13.2 | 19.8 ± 14.8 | 19.7 ± 14.4 |
| Optic Nerve Lt | 43.5 ± 10.8 | 45.5 ± 11.6 | 46.1 ± 11.4 | 46.0 ± 13.9 | 46.2 ± 14.2 |
| Optic Nerve Rt | 29.2 ± 12.4 | 24.2 ± 17.2 | 26.0 ± 17.0 | 19.6 ± 19.2 | 21.6 ± 19.9 |
| Pituitary | 37.9 ± 15.3 | 35.8 ± 18.5 | 36.1 ± 18.5 | 35.6 ± 20.0 | 36.0 ± 20.1 |
| Retina Left | 36.1 ± 15.1 | 38.1 ± 13.9 | 38.3 ± 13.2 | 37.4 ± 14.1 | 38.8 ± 13.6 |
| Retina Right | 13.0 ± 9.2 | 8.7 ± 8.9 | 8.4 ± 9.0 | 9.4 ± 11.0 | 8.6 ± 10.1 |
| Spinal Cord | 22.8 ± 11.9 | 9.4 ± 8.4 | 8.5 ± 7.7 | 8.7 ± 6.6 | 8.5 ± 5.3 |
| Left Temp Lobe | 35.5 ± 24.0 | 37.5 ± 17.3 | 39.0 ± 16.9 | 38.5 ± 18.1 | 39.7 ± 18.0 |
| Right Temp Lobe | 8.4 ± 6.1 | 4.1 ± 6.5 | 4.4 ± 6.3 | 3.4 ± 5.9 | 3.4 ± 5.7 |

Table A13: Variable RBE-weighted dose statistics from the Lyngholm RBE model (LYN) for all five modalities with first standard deviation and colour coded from lowest (blue) to highest (red) dose. MD: mean dose.

|  | VMAT | IMPT | IMPT-LET | Arc | Arc-LET |
| --- | --- | --- | --- | --- | --- |
| Body | 7.5 ± 3.0 | 6.2 ± 2.1 | 6.4 ± 2.2 | 6.4 ± 2.4 | 6.6 ± 2.5 |
| Brain | 5.9 ± 2.7 | 4.3 ± 2.2 | 4.5 ± 2.4 | 4.2 ± 2.0 | 4.5 ± 2.1 |
| Brainstem D10% | 30.9 ± 7.9 | 20.8 ± 16.3 | 19.6 ± 15.1 | 23.1 ± 15.5 | 23.5 ± 15.4 |
| Brainstem D50% | 18.7 ± 10.5 | 7.9 ± 8.4 | 7.2 ± 7.6 | 8.7 ± 7.9 | 9.3 ± 8.5 |
| Brainstem D_0.1cc_ | 40.5 ± 10.3 | 34.6 ± 20.3 | 33.9 ± 19.8 | 38.8 ± 18.8 | 38.7 ± 18.8 |
| brainstemCore | 35.9 ± 9.9 | 25.7 ± 17.8 | 24.3 ± 17.1 | 29.4 ± 17.0 | 29.9 ± 17.2 |
| Cochlea Left | 42.6 ± 12.9 | 40.6 ± 19.8 | 40.3 ± 19.7 | 40.5 ± 21.2 | 36.6 ± 18.6 |
| Cochlea Right | 13.6 ± 11.9 | 12.6 ± 16.4 | 12.7 ± 16.9 | 11.4 ± 16.4 | 14.6 ± 22.5 |
| HippoHead Lt | 12.1 ± 12.8 | 14.3 ± 10.2 | 14.6 ± 10.6 | 15.0 ± 10.5 | 14.3 ± 11.9 |
| HippoHead Rt | 3.4 ± 1.8 | 2.2 ± 2.6 | 2.7 ± 2.8 | 1.4 ± 2.3 | 3.0 ± 5.6 |
| HippoTail Lt | 5.0 ± 6.5 | 3.4 ± 4.6 | 3.5 ± 4.7 | 3.5 ± 4.6 | 3.5 ± 5.3 |
| HippoTail Rt | 2.0 ± 0.9 | 0.2 ± 0.3 | 0.3 ± 0.4 | 0.2 ± 0.3 | 0.5 ± 1.2 |
| Hypothalamus | 3.4 ± 1.9 | 4.0 ± 3.6 | 4.2 ± 4.6 | 3.5 ± 3.1 | 3.7 ± 3.4 |
| Lacrimal Left | 24.0 ± 17.0 | 22.6 ± 16.3 | 23.1 ± 16.4 | 23.2 ± 18.8 | 24.3 ± 18.7 |
| Lacrimal Right | 5.2 ± 4.2 | 1.0 ± 2.0 | 1.2 ± 2.3 | 1.0 ± 1.9 | 1.0 ± 1.7 |
| Lens Left | 12.0 ± 9.4 | 6.3 ± 5.5 | 6.1 ± 5.8 | 7.2 ± 7.8 | 7.0 ± 7.3 |
| Lens Right | 3.7 ± 1.5 | 0.8 ± 0.8 | 0.8 ± 0.8 | 1.1 ± 1.1 | 1.3 ± 1.1 |
| Optic Chiasm | 15.8 ± 9.8 | 20.1 ± 15.1 | 19.9 ± 13.7 | 20.7 ± 15.6 | 20.6 ± 15.0 |
| Optic Nerve Lt | 43.5 ± 10.8 | 48.0 ± 12.3 | 48.5 ± 12.0 | 48.6 ± 14.7 | 46.6 ± 16.9 |
| Optic Nerve Rt | 29.2 ± 12.4 | 25.4 ± 18.0 | 27.1 ± 17.7 | 20.6 ± 20.2 | 24.3 ± 21.3 |
| Pituitary | 37.9 ± 15.3 | 37.8 ± 19.5 | 38.0 ± 19.5 | 37.7 ± 21.3 | 38.0 ± 21.2 |
| Retina Left | 36.1 ± 15.1 | 40.0 ± 14.4 | 40.3 ± 13.8 | 39.3 ± 14.8 | 39.0 ± 17.2 |
| Retina Right | 13.0 ± 9.2 | 9.1 ± 9.3 | 8.9 ± 9.4 | 9.9 ± 11.6 | 10.7 ± 11.2 |
| Spinal Cord | 22.8 ± 11.9 | 9.9 ± 8.9 | 9.0 ± 8.1 | 9.2 ± 6.9 | 9.0 ± 5.6 |
| Left Temp Lobe | 35.5 ± 24.0 | 38.5 ± 17.5 | 40.2 ± 17.2 | 39.5 ± 18.2 | 40.8 ± 18.0 |
| Right Temp Lobe | 8.4 ± 6.1 | 4.3 ± 6.8 | 4.7 ± 6.5 | 3.6 ± 6.2 | 3.6 ± 6.1 |

Figure A3: Delivery times for the boost plan for IMPT, LET-optimized IMPT, PAT and LET-optimized PAT for all patients. The median patient is given by the dashed lines.
